# Control analysis of the eukaryotic cell cycle using gene copy-number series in yeast tetraploids

**DOI:** 10.1186/1471-2164-14-744

**Published:** 2013-10-31

**Authors:** Annette A Alcasabas, Michaela de Clare, Pınar Pir, Stephen G Oliver

**Affiliations:** 1 Department of Biochemistry, University of Cambridge, Tennis Court Road, Cambridge CB2 1GA, UK; 2 Cambridge Systems Biology Centre, University of Cambridge, Tennis Court Road, Cambridge CB2 1GA, UK; 3 Current address: BioSyntha Technology, BioPark Hertfordshire, Broadwater Road, Welwyn Garden City AL7 3AX, UK; 4 Current address: Babraham Institute, Babraham Campus, Cambridge CB22 3AT, UK

**Keywords:** Cell cycle, Control analysis, Copy number variation, Logical model, Tetraploid, Yeast

## Abstract

**Background:**

In the model eukaryote, *Saccharomyces cerevisiae*, previous experiments have identified those genes that exert the most significant control over cell growth rate. These genes are termed HFC for high flux control. Such genes are overrepresented within pathways controlling the mitotic cell cycle.

**Results:**

We postulated that the increase/decrease in growth rate is due to a change in the rate of progression through specific cell cycle steps. We extended and further developed an existing logical model of the yeast cell cycle in order elucidate how the HFC genes modulated progress through the cycle. This model can simulate gene dosage-variation and calculate the cycle time, determine the order and relative speed at which events occur, and predict arrests and failures to correctly execute a step. To experimentally test our model’s predictions, we constructed a tetraploid series of deletion mutants for a set of eight genes that control the G2/M transition. This system allowed us to vary gene copy number through more intermediate levels than previous studies and examine the impact of copy-number variation on growth, cell-cycle phenotype, and response to different cellular stresses.

**Conclusions:**

For the majority of strains, the predictions agreed with experimental observations, validating our model and its use for further predictions. Where simulation and experiment diverged, we uncovered both novel tetraploid-specific phenotypes and a switch in the determinative execution point of a key cell-cycle regulator, the Cdc28 kinase, from the G1/S to the S/G2 boundaries.

## Background

In constructing a systems-level model of the eukaryotic cell, insight can be gained by considering the system from the ‘top down’, and populating the model with only the most important components, i.e. those exerting the greatest degree of control over cellular processes. The approach can be mathematically formalised using Metabolic Control Analysis (MCA) [1,2] which describes how the relationship between pathway fluxes and reagent concentrations depends on the network properties. More recently, the approach has been used to describe not only metabolic, but also signalling networks [3]. The relative degree of control which a given protein product exerts on a pathway is quantified via its *flux control coefficient*:

CEJ=ΔJ/JΔE/E

That is, the *control coefficient C*_*E*_^*J*^ describes the change in the flux *J* at steady state that results from the relative perturbation of an effector *E* and so represents the fraction of control which the particular effector exerts over the total flux. To describe, as we seek to here, the relative importance of given genes within the genome of the model eukaryote *Saccharomyces cerevisiae*, and constrain the value of a given protein’s (*i.e.* effector’s) control coefficient, complementary experiments must be performed: 1. changing the flux through the pathway and measuring the impact upon protein concentration (described in [4,5]); 2. changing the protein concentration and measuring the impact upon the pathway flux.

The latter approach was first taken in our previous experiments using the set of heterozygous deletion mutants for all protein-encoding genes in the yeast genome [6,7]. Through the measurement of growth rates in batch culture, and competition experiments in continuous culture, we identified a set of genes in *S. cerevisiae* for which the heterozygous deletion mutants exhibit a growth rate significantly different from that of the wild type (WT). Together, these *haploinsufficient* (the heterozygous mutant displays slower growth than the WT; HI) and *haploproficient* (faster than WT growth when heterozygous; HP) genes form a set of ‘high flux-control coefficient’ (HFC) genes which, together with the essential genes, exhibit significant control over the cell growth rate and are candidates for inclusion in our ‘coarse-grained’ cell model.

These previous experiments, however, only explored the flux at 100 and 50 percent of WT gene dosage. To be able to study the variation in flux at more intermediate gene dosages, and hence more precisely measure the values of the control coefficients, we analysed the effect on growth of sequential deletions of alleles in *tetraploid* yeast cells. With four copies of the genome in the tetraploid, we are able to construct mutants having 0, 25, 50 and 75% of WT gene dosage (corresponding to 0, 1, 2 and 3 copies of the gene, respectively) for each of eight HFC genes comprising two signal transduction pathways controlling the G2/M cell cycle transition. In doing so, we followed the approach taken in a previous study, which measured flux control coefficients for the tryptophan biosynthetic pathway [8]. Allowing an increased range of flux variation, without employing either overexpression or non-native promoters, the tetraploid system is ideal for the determination of flux control coefficients. However, widespread adoption of the approach has been frustrated by the difficulty of constructing tetraploids, and we believe this paper reports the first systematic study of tetraploid series for pathway analysis since the original work by Niederberger *et al.*[8].

**Figure 1:**
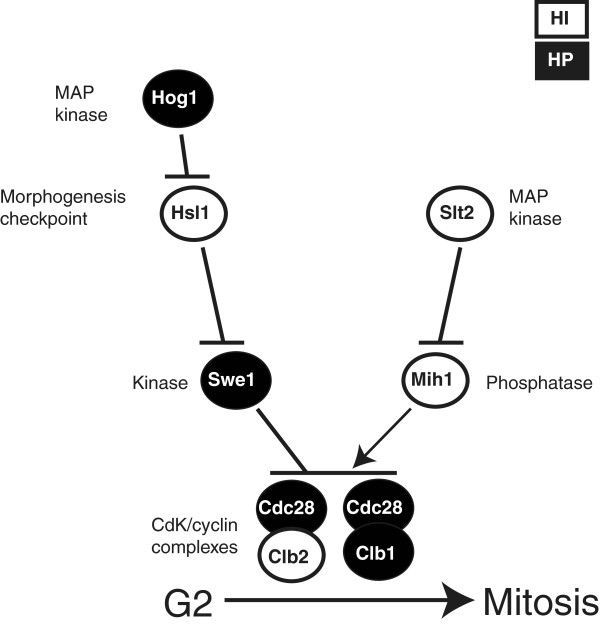
**Regulation of the G2/M transition in the yeast cell cycle.** Diagram of the eight gene products which form the checkpoint module linking the osmotic stress response (through the Hog1 MAP kinase) and the cell wall integrity pathway (through the Slt2 MAP kinase) to the regulation of the cell cycle G2 to mitosis transition.

Several cellular processes, such as transcription and translation, are particularly enriched for high-flux-control genes. For instance, genes encoding subunits of the 80S ribosome are strongly haploinsufficient, while those encoding components of the general transcription factor TFIID complex are enriched for haploproficiency [7]. The *S. cerevisiae* cell cycle, too, is enriched for HFC genes [9], and we propose that the deviation from the wild-type growth rate observed in heterozygotes is due to the cell progressing through the cell cycle at an accelerated/decelerated rate. Thus, studying the growth rate phenotype is equivalent to studying the cell cycle period. In the present work, in order to study the interaction of the growth phenotypes and cycle periods of HFC genes, we focussed our tetraploid studies on a cell cycle checkpoint module for which each member is encoded by an HFC gene (Figure 1). The module consists of two mitogen-activated protein kinase (MAPK) cascades enriched for HFC genes: the cell wall integrity (CWI) and the high osmolarity glycerol (HOG) pathways, which co-regulate mitotic entry in response to stresses such as temperature and osmolarity. The pathways converge on the complex of the cyclin-dependent kinase Cdc28p and the G2 cyclins Clb1p and Clb2p, the presence of which are required to drive the G2-to-M phase transition. These pathways represent two of at least six distinct *S.cerevisiae* MAP kinase cascades which control the regulation of gene transcription in response to extra- and intra-cellular stresses and signals. They are highly conserved, both structurally and functionally, among eukaryotes [10,11].

The first cascade, the CWI pathway, controls the process of cell wall synthesis as the cell changes volume and shape during the cell cycle and in response to heat and hyperosmotic stress or cytoskeletal perturbation [11,12]. These stresses are sensed by different components of the pathway and result in activation of the Slt2p kinase [13] and the eventual remodelling of the cell wall. When activated, the pathway also contributes to G2/M cell cycle arrest. Genetic evidence suggests that this occurs by blocking the activity of the Mih1 phosphatase [14], which is required to remove inhibitory phosphorylation on tyrosine 19 of Cdc28p. In contrast, the cell responds to *hyper*osmolarity by activating the high osmolarity glycerol (HOG) pathway, which leads to a higher intracellular glycerol concentration to restore the osmotic gradient. Elevated glycerol production is mediated by the Hog1 kinase and involves both increased gene expression and a metabolic response [15,16]. In addition, activation of this pathway leads to transient cell cycle arrest until the right glycerol concentration is reached. Hog1p-mediated arrest at the G2/M transition occurs, in part, by triggering the morphogenesis checkpoint. Hog1p phosphorylates Hsl1p [17], which (when a bud is correctly formed) is present at the bud neck and recruits and degrades Swe1p [18,19]. The Swe1p kinase negatively regulates Clb2p-Cdc28p activity by binding to the complex and phosphorylating Cdc28p on tyrosine 19 [20]. The morphogenesis checkpoint thereby works to delay mitotic entry until a critical bud size is reached, and defects in the actin cytoskeleton or septin assembly are overcome. Cells arrested by the checkpoint are significantly elongated since polarised growth persists during budding, instead of isotropic growth resuming within the bud [21]. In contrast, overriding the checkpoint interferes with correct nuclear segregation and results in an accumulation of binucleate mother cells [18,22].

## Results

### Copy number variation affects growth rate, viability, and cell cycle progression, and *in silico* simulations are predictive of *in vivo* behaviour

In an attempt to predict the trends in phenotypic variation that we might observe in the tetraploid mutant series, we constructed a logical model of the *S.cerevisiae* cell cycle and simulated the effects of deleting the HFC G2/M checkpoint genes. Our model is based on that of Fauré *et al.*[23], which incorporates 27 genes and 4 cell-cycle states as nodes, the Boolean state of which is governed according to logical expressions updated in discrete, equal-length, time-steps. The Fauré *et al.* model is, in turn, an adaptation of an earlier differential-equation cell cycle model [24].

**Figure 2:**
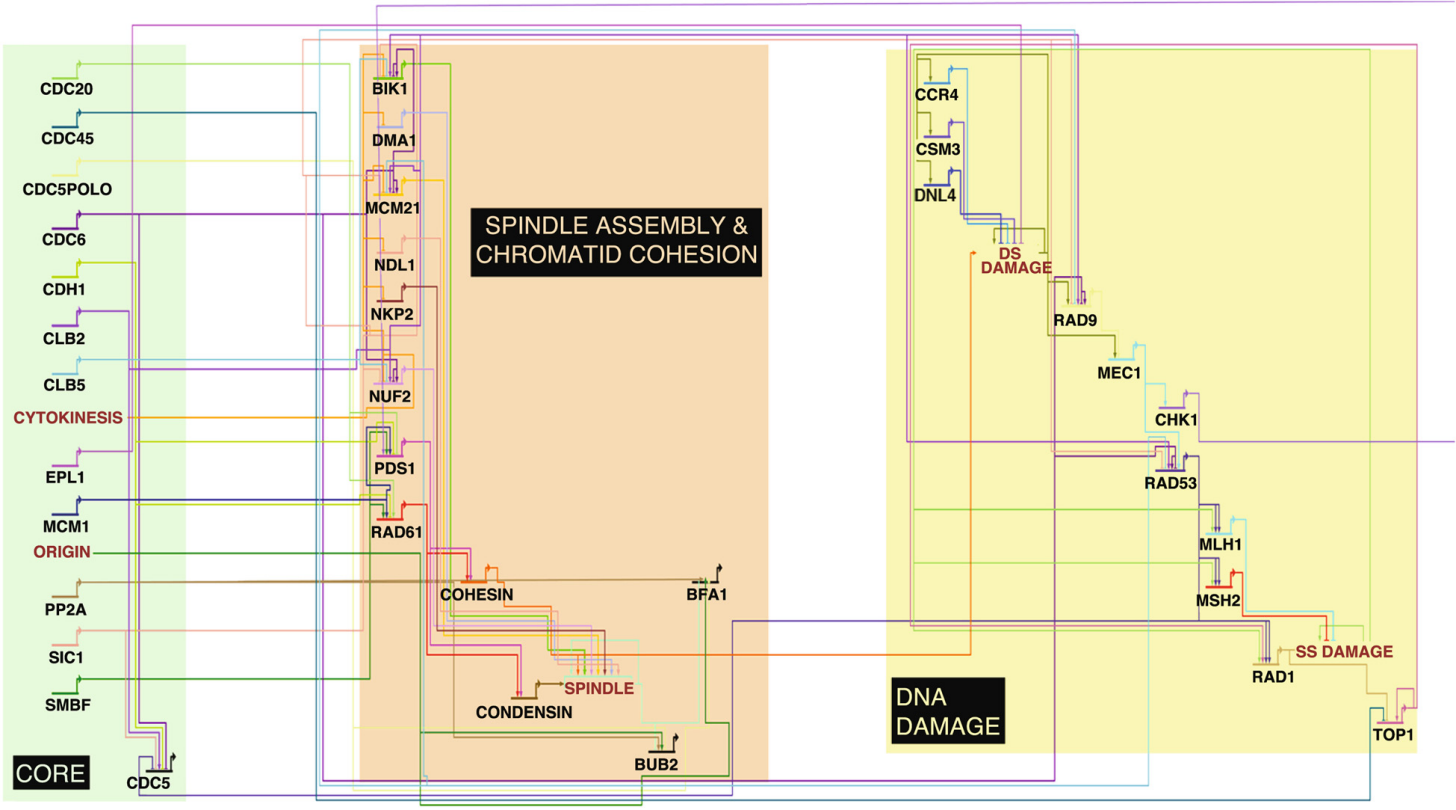
**Extended model of the yeast cell cycle.** New modules added to the logical model of the yeast cell cycle developed by [23], represented in BioTapestry [26].

We extended the Fauré *et al.* model, adding a further 30 cell-cycle-related genes (Figure 2 and see Additional file 1: Table S1 and Model S1), all of which had been identified as HFC in our diploid screen [7]. The logical relationships linking in the new nodes were inferred from published data on the wiring of the *S.cerevisiae* cell cycle [25]. We also added several further self-contained modules (representing the spindle checkpoint, and the DNA damage-response checkpoint), which can be selectively incorporated into simulations. The model states are associated, as in Fauré *et al.*[23], with cell cycle stages. Briefly, G1 begins at START with the activation of the G1 cyclins and their associated species, cell mass being at its lowest level, and the cell in an unbudded state. Levels of the Cdc28p*/*Clb complex are low, due to the presence of B-type cyclin inhibitors, and the genes in the morphogenesis checkpoint (the species we have studied here) are not active, due to the lack of a bud. S and G2 phases are characterised by the degradation of the B-type cyclin inhibitors and the consequent accumulation of Clb1p and Clb2p, the emergence of a bud, and the subsequent activation of the morphogenesis checkpoint genes. The relevant hallmarks of M phase are the completion of the budding process, and thus the inactivation of the morphogenesis checkpoint and the gradual decline of B-type cyclin levels as the cell proceeds through the exit from mitosis following cytokinesis. Using the signature features thus defined, the number of time-steps the simulation spends in each cell cycle phase may be counted and, consequently, a cell cycle profile assembled. Profiles for each of the deletion strains were calculated by counting the number of time-steps, out of 10,000, spent in each stage. Results were averaged over the 10,000 simulations, and profiles were normalised to the WT profile, giving the relative proportions of cells in G1, G2/S and M.

**Figure 3:**
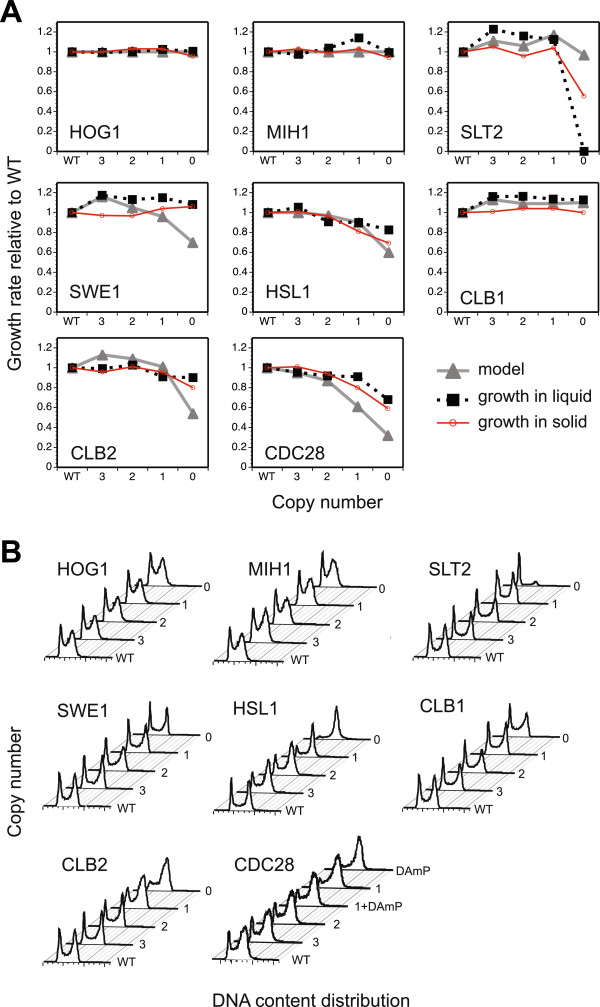
**Comparison of model prediction and *in vivo *data for tetraploid growth rates and cell-cycle distributions. a)** growth rates relative to wild type for the strains within each of our tetraploid deletion series; experimental data (in liquid medium: black dashed lines, squares; on solid medium: red solid lines, circles) is compared to model predictions (grey solid lines, triangles); **b)** cell cycle distributions of the tetraploid deletion series measured by flow cytometry; proportion of cells with G1 (leftmost peak) and G2/M (rightmost peak) DNA content are shown.

**Figure 4:**
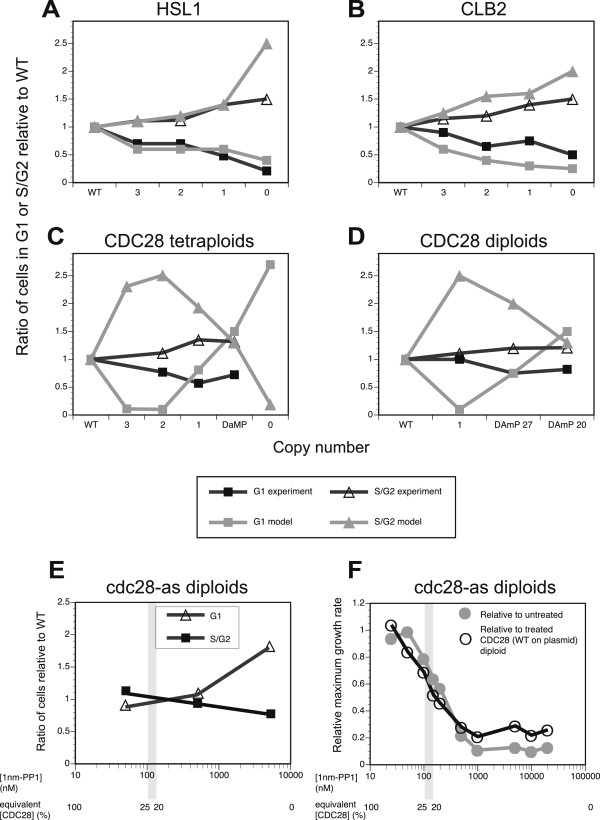
**Comparison of model predictions and imaging flow-cytometry measurements of the quantitative cell cycle profiles. a)***HSL1,***b)***CLB2,***c)***CDC28* tetraploid deletion series; **d)***CDC28* diploid series. Proportion of cells relative to WT in G1 (experiment: grey squares; model prediction: black squares) and S/G2 (experiment: grey triangles; and model prediction: black open triangles). **e)** Relative accumulation in the G1 or G2 phase of the cell cycle for the diploid *cdc28/cdc28 + cdc28-as1* strain with increasing inhibitor concentration, **f)** Effect of increasing concentrations of 1 nm-PP1 specific inhibitor on the growth rate relative to the untreated *cdc28/cdc28 + cdc28-as1* strain and to *cdc28/cdc28 + CDC28* treated with the same inhibitor concentration, with mRNA level/growth rate relationships from the tetraploid series indicated for comparison.

A novel feature of our extended model enables its use to predict the phenotypic effect of gene copy number variation. The extended model’s functionality allows it to simulate the deletion of a subset of copies of a given gene, by defining the probabilities with which these species are located within each logical expression (see Methods). A similar mechanism allows us to simulate the effects of inhibitors, DNA-damaging agents, or osmotic stress. The model displays a robustness similar to that of the original model of Fauré *et al.*[23]. WT cell cycle progression is similar, though progression times for different stages are altered due to the presence of additional, often intermediate, gene products. Using this model, we predicted both the cell-cycle period (and, consequently, growth rate; Figure 3a) and progression (Figure 4 & Additional file 1) for each member of the tetraploid series by simulating mutants having 0, 25, 50 and 75% of WT gene dosages (hereafter referred to as null-, single-, two- and three-copy, respectively). The model also allows us to identify the precise mechanisms underlying phenotypic changes, since we can follow the relative amount of time spent at each step of the cell cycle and note the particular nodes whose activation/inhibition is compromised in the deletant relative to the wild type. It is important to emphasise that, in simulations employing the model, time is measured in fractions of a cell cycle, rather than chronological time. This biological measure of time is probably more relevant to the study of gene-dosage effects since growth rate, and hence the cycle time in minutes, can alter according to the identity or supply of specific nutrients or other physiological factors.

### Construction of tetraploid series for HFC genes

We created tetraploid deletion series for each of the eight genes under consideration by mating the relevant mating-competent diploid deletion mutants, as described in Methods. We believe that this approach represents a powerful tool in probing flux through metabolic and signalling pathways to a more precise degree than traditional diploid/haploid screens. Using RT-PCR, we have quantified the effect of incrementally deleting the copies of a gene in the tetraploid on both the target gene mRNA levels, and those of the geneticin-resistance kanMX cassette with which it was replaced (see Methods). These determinations confirmed that, for each of the eight tetraploid series, deletion of all four copies completely ablated gene expression and, for the intermediate copy-number strains, the gene mRNA levels approximate the expected 25, 50 and 75% reductions compared to those measured in the WT tetraploid (see Additional file 1).

Tetraploid yeast strains have been reported to have less stable genomes than diploids or haploids [27,28], particularly over long periods in stationary phase [29]. Any chromosome loss would render the series non-isogenic and introduce confounding factors, such as the change in dosage of many other genes, and would impact on the measured growth rate of the strain. To avoid this, we measured the DNA content of all tetraploid strains prior to making frozen stocks, all pre-cultures were inoculated directly from such stocks, and sub-cultured only once. All analyses were performed in YPD medium and no batch culture was kept for more than 48 hours. Our flow cytometry results (Figure 3b) demonstrate that no reduction in DNA content occurred under these experimental conditions. To further underscore the genomic stability of our tetraploids in exponential phase, we grew a pool of strains in chemostat continuous culture in a complex, but chemically defined, medium [30] for a period of 4 days (30 to 35 generations) and observed no reduction in DNA content (data not shown).

### Exploiting tetraploid series to probe the impact of gene dosage on cell cycle phenotypes

#### *HOG1* and *MIH1* mutants display no phenotype in the absence of external stress, but the *HOG1* deletion series displays a non-linear response to osmotic stress

In both simulations and experiments, variation in *HOG1* gene copy number did not affect the growth rate (Figure 3a). Strain viability was similar to that of the WT (Additional file 1), and incremental deletions of the genes also had no effect on cell cycle progression (Figure 3b). Similarly, *MIH1* reduction in the tetraploid had no effect on either growth rate or cell cycle distribution, consistent with previous results for *mih1* null haploids [31] and, as for *HOG1*, the *MIH1* simulations agree with our experimental observations. This lack of phenotype is to be expected, as the osmolarity signalling pathway should be inactive in the absence of osmotic stress.

**Figure 5:**
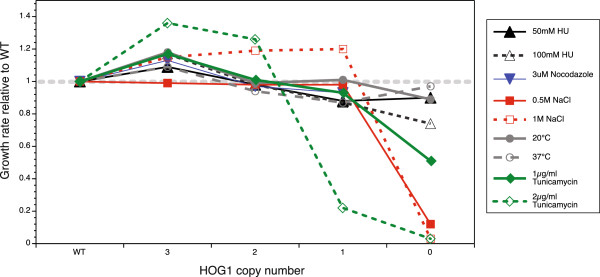
**Response of the *HOG1 *deletion series to a range of stress treatments, showing the resistance of the individual deletion mutants.** Liquid growth profiling, fitness measured as the growth rate in the stressed condition relative to untreated growth, relative to the WT.

Stress treatments, however, elicited significant phenotypic responses in the *HOG1* series. In particular, we observed a consistent, non-linear relationship between salt stress tolerance and *HOG1* gene dosage: the three-copy tetraploid is significantly more resistant to stress than the WT (median *p-*value across stresses = 0.007, one-sided t-tests); the two-copy mutant is equally as fit as the WT (average *p-*value for deviation from WT growth = 0.14), and the null mutant is typically more sensitive (median *p-*value = 0.01, one-sided t-tests; Figure 5) in both liquid and solid cultures. The *hog1* null tetraploid is roughly twice as sensitive as the intermediate-dosage strains at 0.5 and 1 M NaCl (Figure 5) and inviable at 2 M (data not shown), as are the null diploid and haploid mutants [32,33].

This suggests that the *HOG1*-coordinated response to stress in the WT may, in effect, be an ‘overreaction’ in that *S.cerevisiae* can tolerate stresses with less than WT levels (in this case, as low as 25% of the WT level) of Hog1p activity. The decreased Hog1p levels allow the cell cycle to proceed, rather than arresting it during the stress response. The *HOG1* pathway is at the core of the *S.cerevisiae* stress response, and we hypothesise that it may have evolved to be hyper-responsive, given the extreme stresses to which yeast is exposed in the wild. This concurs with the findings of [34] and [35] who have shown that the degree of up-regulation of the *HOG1* pathway cannot keep pace with a ‘ramped’ increase in osmotic stress. We suggest that the cell may compensate for this failing by overreacting on first exposure to salt stress, with the result that the initial pathway activation level might be sufficiently high to manage a ramp in stress until the Hog1 response can be recalibrated. This is also consistent with previous studies showing that a metabolic response to osmotic stress occurs immediately and is essential, while the Hog1p-mediated transcriptional response is secondary [16,36]).

Additionally, the *hog1/hog1/hog1/hog1* null tetraploid is highly sensitive to the ER-stress and G1-arrest–inducing [37] agent tunicamycin (Figure 5). This is congruent with haploid mutant results (deletion sensitises cells, whilst overexpression confers resistance; [38]) and supports the idea that Hog1p may be required for proper G1 progression. In particular, *HOG1* may be implicated in the synthesis of G1 cyclins on exit from G0 [38], although *HOG1* is also implicated in the late stages of the ER stress response, where it is thought to stabilise the autophagy protein Atg8p [39].

### *SLT2* depletion severely compromises growth in the tetraploid

Modelling and experimental results are in quantitative agreement for mutants having at least one copy of the *SLT2* gene (but fewer than the WT) – strains grow significantly faster (by ~20%) than WT (Figure 3a). Both the *in vitro* and *in silico* cell cycle profiles of the partial *slt2* deletants are comparable to the WT – suggesting, interestingly, that the increased growth rate is not due to the cell speeding through one particular cell cycle step, as we observed in other mutants, but rather that *all* stages (or, at least, both G1 and G2; Figure 3b) proceed at an accelerated rate. As well as the G2/M transition we have studied here, the *SLT2* pathway targets the heterodimeric SBF (Swi4-Swi6 cell-cycle box binding factor) transcription factor involved in the G1/S transition [12]; the *slt2* deletion appears to be simultaneously affecting both transitions to produce this effect.

Experimentally, we made the novel observation that the complete absence of *SLT2* severely compromises fitness in the tetraploid state. The tetraploid null mutant is very slow growing, forming small colonies on solid medium, and failing to achieve exponential growth after 20 hours in liquid medium; whereas the diploid null mutant is viable. The strongly fitness-compromised tetraploid null mutant population consists almost entirely of cells arrested in G1 (Figure 3b), suggesting that either the *SLT2* pathway impacts upon the G1/S more strongly than the G2/M transition or that these cells have a very slow exit from the previous stationary phase.

**Figure 6:**
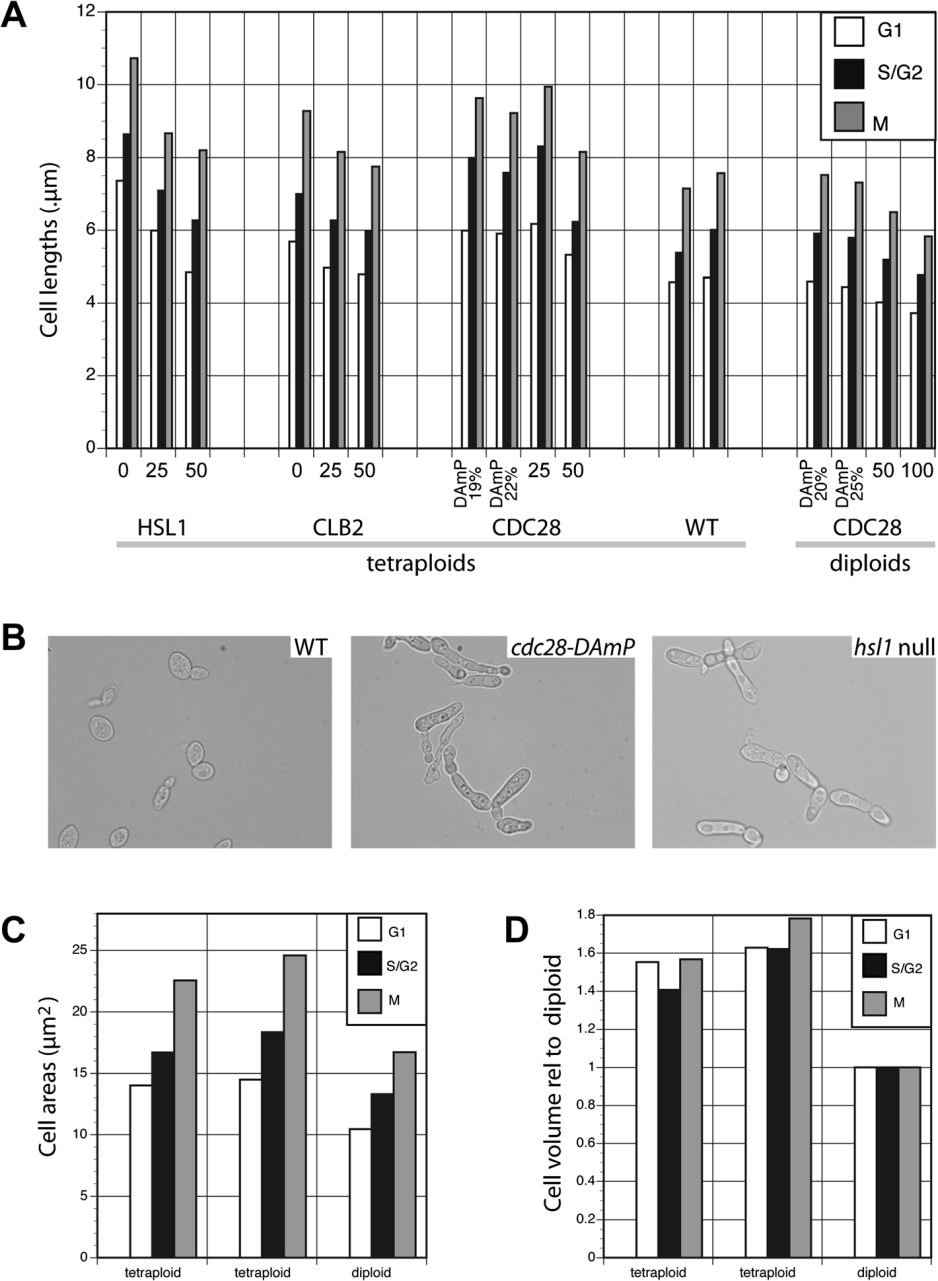
**Cell morphology of the tetraploid series. a)** Cell lengths of the *hsl1, clb2, cdc28* mutant and WT tetraploid strains and *cdc28* diploid strains, plotted against percentage gene dosage, G1 cells (white bars), S/G2 phase cells (grey bars), Mitotic cells (grey bars); **b)** images of WT, *cdc28-DAmP* and *hsl1* null tetraploid strains; **c)** cell areas of WT tetraploid and diploid strains; and **d)** projected volumes of WT tetraploids relative to WT diploids. Measurements from two independently isolated WT tetraploid strains are shown in a, c, and d.

In contrast to what we observed for tetraploids, null *slt2* haploids and diploids are completely viable. This suggests a tetraploid-specific basal level of activation of the Slt2p-mediated cell wall integrity (CWI) pathway. Consistent with this, we found that the slt2 null tetraploids completely fail to grow when subjected to heat (37°C) or cold (21°C) stresses that activate the CWI pathway (data not shown). It has been suggested that the decreased ratio of cell surface area to cell volume in tetraploid cells leads to altered regulation of cell surface components [40]. While cell size measurements using imaging flow cytometry show that the cell volume of the WT tetraploid is roughly 60% larger than that of the WT diploid, this corresponds to a cell surface area increase of only 36% (Figure 6c,d). In the study by Wu *et al.*[40], tetraploids were compared to isogenic haploids and it was found that the increased cell size leads to differential expression of genes encoding components of the cell wall, plasma membrane, extracellular matrix, and their intracellular regulators. These transcriptional changes are partly attributed to an increase in MAPK signalling through the mating and filamentation MAPK pathways, the results also showed that the *SLT2* mRNA is two-fold more abundant in tetraploids than haploids [40].

We therefore subjected isogenic WT haploids, diploids, and tetraploids to a number of cell-wall-specific stresses (sorbitol, calcofluor white, and salt). Tetraploids were no more sensitive to sorbitol and calcofluor white than the isogenic diploid or haploid strains (see Additional file 1). However, we did find that WT tetraploids are more sensitive to 1 M salt than WT diploids (data not shown). This suggests that, like the Slt1p-mediated MAPK pathway, the Hog1p pathway may also be constitutively active at a low level in tetraploids, which can also explain the overreaction to salt stress by WT tetraploids, discussed above, which was not observed with WT diploids (data not shown).

### *SWE1* deletion mutants exhibit faster growth

For *SWE1,* reduction in copy number from four (WT level) to three, resulted in an approximately 18% increase in growth rate (Figure 3a). Growth remained relatively constant at this increased rate upon further reduction in copy number, and viability remained comparable to the WT in all cases (Additional file 1). However, whilst the model’s results for *swe1* deletions predict a similar increase in growth rate for the three- and two-copy mutants, the simulated growth rate subsequently decreases with decreasing copy number (*i.e.* for the single-copy and null mutants). Since the tetraploid phenotype is congruent with *SWE1* haploproficiency in the diploid, the fault seems to lie with the model, rather than any difference between the diploid and tetraploid cell cycles.

### Deletion of *HSL1* affects growth

For *HSL1,* both *in vivo* and in the model’s simulation, growth rate decreased monotonically with successive deletions, with the null mutant achieving only ~65% of the WT rate (Figure 3a). The lower copy number strains exhibit significantly decreased viability, with PI staining indicating death rates of up to 10% of the population compared to 1.5% for the WT (see Additional file 1). Further reductions in copy number result in increasing numbers of cells with a G2 DNA content (Figure 3b). Imaging flow cytometry on an asynchronous culture of the *hsl1* null tetraploid showed that the combined proportion of S-phase and G2 cells in the population is 51%. This is about 1.5 times that found in WT tetraploids (35% of cells in S phase and G2; Figure 4a and Additional file 1) and is in excellent quantitative agreement with our modelling results. Furthermore, the null tetraploid is sensitive to treatment with hydroxyurea (data not shown), which inhibits ribonucleotide reductase and so causes S-phase arrest [41]. This is consistent with the phenotype of *hsl1* haploids, which show a very slow recovery from HU-induced S-phase arrest due to the inefficient degradation of accumulated Swe1p [42].

We had previously identified *HSL1* as haploinsufficient, and the growth rate of the tetraploid two-copy mutant (approximately 90% of the WT rate) is comparable with our results for the growth of the heterozygous diploid deletion mutant. Imaging flow cytometry also demonstrated that single-copy and null *hsl1* tetraploids display an abnormal, elongated, morphology (Figure 6a and 6b) similar to that reported for null haploids in certain genetic backgrounds [21]. In other strains, the elongated morphology and G2 accumulation phenotypes of *hsl1* null haploids are only seen in combination with modest overexpression of Swe1p [18]. These findings suggest that the G2/M transition is very sensitive to the balance between Swe1p and Hsl1p and that the critical level of Hsl1p is above a single copy (>25% gene dosage) in the S288C-derived tetraploid strain.

### Phenotypes of the B-type cyclin deletions differ significantly

A particularly significant prediction that emerged from our initial modelling was the divergent phenotypes of deletions of the two B-type cyclin genes, *CLB1* and *CLB2*, particularly at the lowest copy numbers (*i.e.* in the null and the single-copy mutants). This prediction emerged directly from the fact that, despite being otherwise wired into the cell cycle identically, *CLB2* degradation by the APC/C begins earlier than degradation of *CLB1* (reviewed in [43]), hence the response to reducing the *CLB2* copy number is more pronounced. Model predictions were subsequently confirmed experimentally, with the measured growth rates in excellent quantitative agreement with our simulation results (Figure 3a).

We previously identified *CLB1* as haploproficient and *CLB2* as haploinsufficient in diploid cells, and our tetraploid findings are consistent with this. Any partial or full deletion of *CLB1* leads to a significantly faster growth rate (approximately 20% faster; Figure 3a) than WT, with strain viability comparable to that of WT. For *CLB2,* the three- and two-copy tetraploids have a growth rate similar to WT. However, at the single-copy and null *CLB2* gene dosages, cells exhibit a significant reduction in both viability and maximum growth rate due to a delay in the G2/M transition (Figures 3b and 4b; Additional file 1). This suggests that there exists a threshold level, probably between the single- and two-copy gene dosages (*i.e.* 25-50% WT dosage), below which the concentration of the *CLB2* protein product cannot drop if proper cell function is to be maintained. In this case, the haploinsufficiency of *CLB2* in the diploid would be due to the heterozygous deletion taking the concentration below this threshold dose. This suggests that the effect upon protein levels of deleting one copy in the diploid is more severe than that of deleting two of four copies in the tetraploid. It should be noted, however, that the simulated null mutant is slightly more unfit than is observed *in vivo*. Moreover, the simulated variation in the cell-cycle profile due to the G2/M delay is also more extreme than that observed *in vivo* (Figure 4b).

Flow cytometry demonstrated that, whilst the single-copy and null *clb2* deletion mutants suffer pre-mitotic cell cycle arrest, deletions of *CLB1* have no effect on cell-cycle progression (Figure 3b). The contrast in phenotypes is consistent with observations that, among the four mitotic cyclins (Clb’s 1 to 4) in budding yeast, Clb2p plays the major role at mitotic entry [44,45]. Clb2p uniquely localises to the bud neck during mitosis [46], where it is required for the degradation of Swe1p [47]. Mutations that prevent this localisation result in G2/M delay and in cells with an elongated morphology. This exactly corresponds to the phenotype in our tetraploid deletant series (Figure 6a). All this is consistent with the major function of *CLB2* being within the mitotic cell cycle, whilst *CLB1* plays the more significant role in meiosis [48]. *CLB1* is expressed mitotically [44] and the faster cell cycle phenotype we observe is consistent with the idea that more Clb2p-Cdc28 complexes are formed when the level of Clb1p, competing with Clb2p for Cdc28p binding, is reduced.

### CDC28

#### The simulated cell cycle arrest phenotype shows a bimodal transition, dependent on *CDC28* dosage

Cdc28p*,* the primary cyclin-dependent kinase in *S.cerevisiae*, is integral to the regulation of both the early and late stages of the cell cycle. Therefore, we expected variation in *CDC28* copy number to result in pronounced, and potentially more complex, phenotypes than the other HFC genes involved in the G2/M checkpoint. Our simulations predict just this, with incremental deletions of the gene resulting in a severe and sudden growth impairment. Additionally, the simulated cell-cycle profile exhibited a bimodal transition (Figure 4c). As gene dosage decreases from WT levels to 50% of WT, increasing numbers of cells accumulate in G2, reaching a maximum of ~2.5 x WT numbers in the two-copy deletant. The relative number of cells in both the G1 and M phases exhibits a commensurate decline, the former being more pronounced than the latter. The simulations predict that inefficient spindle formation is the major contributing factor to the slowed progression through G2. This is consistent with experimental observations in haploid cells bearing *cdc28* conditional mutations that are arrested in G2, which are defective in spindle pole-body separation [49,50].

Within the model, as gene dosage continues to decrease, the relative number of cells accumulating in G2 begins to *decrease* from this maximum, returning back to WT levels at approximately 20% dosage. Simultaneously, the relative number of cells in G1 begins to rise, returning to WT levels at single-copy gene dosage, until (at the lowest gene dosages) the population is almost completely arrested in G1. The transition in the cell cycle phase in which cells accumulate reflects the changing relative impact of Cdc28p abundance upon G1 and G2 progression as the copy number of the cognate gene is varied. At the very lowest copy numbers, there does not appear to be enough of the kinase available to allow cells to proceed through G1 and initiate DNA replication. Above a certain threshold (roughly one quarter of WT gene dosage), this impediment is overcome and cells can enter into G2. Thereafter, from dosages from one-quarter of WT to the WT-level, cell cycle progression depends on the ability to pass through G2.

### Experiment concurs with simulation but is limited by the lowest viable *CDC28* dosage

As a key cell cycle regulator, *CDC28* is essential in haploids, diploids, and tetraploids (data not shown). Consequently, whilst we were able to construct tetraploids with one, two and three gene copies, we could not obtain the complete (quadruple) deletant. Therefore, to achieve a lower concentration of Cdc28p, we constructed a tetraploid with a single *cdc28*-*DAmP* allele. This allele contains no mutation in its coding region but, owing to the insertion of an antibiotic resistance marker at the immediate 3′ end of the coding sequence, its mRNA is unstable [51]. In two independent isolates of the *cdc28*-*DAmP* tetraploid, we measured the *CDC28* mRNA concentration as 19% and 22% that of WT (see Additional file 1). We also constructed a tetraploid with one wild-type copy of *CDC28* and one copy of *cdc28*-*DAmP*, corresponding to a *CDC28* mRNA concentration of 36% (Additional file 1).

Experimentally, reduction in *CDC28* gene dosage resulted in progressively slower growth rates, reaching a minimum of 60% of WT growth at the lowest dosage (the DAmP allele-only strain). This was the greatest growth-rate reduction observed for any of the genes studied other than the quadruple deletion of *SLT2.* Whilst the simulated drop-off in growth is more pronounced, a marked increase in cell death was also observed in experiments: only 90% of the population is viable (see Additional file 1). This may account for the quantitative disparity between model and observation, since cell viability cannot be taken into account in the simulations. This experimentally observed decline in viability is consistent with the essentiality of *CDC28*; however, the decrease in tetraploid growth rate as gene dosage is reduced is in direct contrast with our observations that the gene is haplo*proficient* in the diploid.

At the lowest *CDC28* expression levels that we were able to obtain (*i.e.* ~20% mRNA dosage in the *cdc28-DAmP*-only strain), cells accumulated with 8C (i.e. 2 N) DNA content. Through imaging flow cytometry, we identified these populations as pre-mitotic, S/G2 cells (Figure 4c). The greatest degree of accumulation occurred in the DAmP allele-only strains, where S and G2 cells accounted for 46% of the population, in contrast to 35% for the WT (see Additional file 1). This suggests that these strains cannot efficiently transit from G2 to mitosis. These observations were recapitulated in flow cytometry results for a diploid series of *CDC28* mutants, *i.e.* a heterozygous diploid *CDC28/cdc28* strain (50% dosage), and a single copy of the *cdc28-DAmP*/*cdc28* diploid (producing 20% of WT mRNA levels; Figure 4d and Additional file 1). The tetraploid deletion series exhibited increasingly elongated cell morphologies with decreasing gene dosage (the *cdc28-DAmP*/*cdc28* having a cell length 33% greater than that of WT; Figure 6a and 6b), a phenotype also observed for the *cdc28-DAmP*/*cdc28* diploid (approximately 20% longer than WT cells; Figure 6a).

The model also predicts a population enriched in G1-phase cells for *CDC28* dosages lower than 20% (Figure 4c and 4d) when, we hypothesise, the level of Cdc28p goes below the G1/S threshold as discussed above. We were not able to verify this experimentally because we could not isolate a *cdc28-DAmP* diploid or tetraploid with a *CDC28* mRNA concentration below 19%. We created both *cdc28-DAmP* diploid and tetraploid strains by plasmid shuffling, with one mating cell carrying the WT *CDC28* gene on a plasmid. We observed a very low rate of *CDC28* plasmid loss for the resulting strains, suggesting that having 19% of the WT *CDC28* transcript level is very close to the threshold required for cell survival.

### G1 arrest occurs at very low Cdc28p activity levels

To generate levels of Cdc28p activity below 19% of WT, we created a diploid *cdc28/cdc28* yeast strain bearing only the mutant *cdc28*-as1 (F88G) allele [49] on a centromeric (single-copy) plasmid. This allele is sensitive to the C3-1′-naphthyl PP1 (1 nm-PP1) derivative of PP1, the Src-family-selective inhibitor [52], which can bind only to the mutant Cdc28p, and not to any other (wild-type) protein kinase. This allows the Cdc28p activity in the strain to be titrated by varying the concentration of inhibitor added. The effect upon the strain’s growth rate of increasing concentrations of 1 nm-PP1, is shown in Figure 4f. Comparing the growth rate at a given concentration to that of the tetraploid deletion series allowed us to estimate the degree of Cdc28p activity present in each strain (Figure 4e).

Cell cycle profiling of this diploid mutant at high (μM) concentrations of inhibitor therefore allowed us to probe Cdc28p activities lower than could be obtained in the tetraploid series (as discussed above), and confirmed the model predictions. Cells accumulate in G2 upon treatment with low concentrations of inhibitor (50 nM) (Figure 4e). Based on the growth rate comparison, this inhibitor dosage reduces Cdc28p activity to levels equivalent to 25% of WT, and the degree of G2 accumulation (126% compared with WT populations) is in agreement with that observed for both diploid DAmP strains having 20-27% of WT mRNA levels and for the single-copy tetraploid strain (Additional file 1). However, at higher inhibitor concentrations (500 and 5000 nM), for which the growth rate indicates that Cdc28p activity is reduced to well below 20% of the WT level, cells arrest in G1 (Figure 4e). Furthermore, the *degree* of G1 accumulation (180% of WT levels at 5000 nM) is in agreement with our simulation predictions.

Our stress-treatment data also support the bimodal Cdc28p dose–response model. Significant sensitivity or resistance to an arrest agent relative to the WT should identify the precise stage within the cell cycle that variation in the dosage of a gene exerts its effect. This is the dosage-variation equivalent to the ‘execution point’, which was defined by Hartwell [53] as the first step at which complete loss of gene function disrupts cell cycle progression. Haploid *CDC28-DAmP* knockdown mutants are sensitive to tunicamycin (which causes G1 arrest) [37]; but our triple *cdc28/cdc28/cdc28/CDC28* deletion mutant is more resistant to the drug than the WT (see Additional file 1). This suggests that there may be a phenotype transition, dependent on levels of *CDC28* below the ~25% of WT levels in the triple-deletion tetraploid. This, combined with the lack of a phenotype for *cdc28* deletants under nocodazole treatment (which causes G2 arrest by microtubule depolymerisation; see Additional file 1), would support our model’s predictions that very low *CDC28* copy numbers may impact more strongly upon G1 than G2.

### Accumulation in G2 in the *CDC28* deletion series is not due to DNA damage

We sought to explore alternative causes of G2 arrest in the *CDC28* deletion series. In particular, several mechanisms exist by which *S.cerevisiae* cells arrest cell cycle progression in response to DNA damage (reviewed in [54]). Using a modified version of our cell cycle model that incorporates the probability of occurrence of different forms of DNA damage, and the sensing and repair pathways [9], we tested whether (*in silico*) deletion of *CDC28* activated the DNA damage response and whether, conversely, *cdc28* deletants were significantly sensitive to disruption of this response. Simulation results suggest that the G2 arrest is independent of the DNA damage response. To confirm this experimentally, we searched for transcriptional up-regulation of damage-response genes by using RT-PCR to determine the levels of their transcripts. The mRNA levels of *DUN1, DUN7, RAD54, PLM2* and *RNR3* (the downstream reporters suggested in [55]) were unchanged from WT levels in either deletion series (see Additional file 1).

## Discussion

Using a combined modelling and experimental approach, we have characterised the degree of control over cell cycle period/growth rate for a complete checkpoint module within the *S.cerevisiae* cell cycle. In the majority of cases, the model predictions and observed phenotypes in our tetraploid series were consistent with previous findings in diploid and haploid strains, validating the current work as an extension of our previous study of high flux-control genes [6] and pathways [7], and emphasising the significance of gene dosage variation rather than ploidy-specific effects. In particular, tetraploid series of *SWE1, CLB1, CLB2* and *HSL1* had similar phenotypes to diploid mutants. These four genes exert a high degree of flux control over the cell cycle, and their sequential deletion had significant effects on growth rate.

This present work looks at the effects of decreasing the gene dosage of 8 cell cycle genes and complements a previous study that looked at the upper limit of gene dosage for 30 cell cycle genes in haploids [56]. Of the five genes that were used in both studies, Moriya and colleagues found that *SWE1, CLB1, CLB2* had a low limit of viable overexpression (<20 copies). In contrast, *CDC28* gene dosage was also limited but to higher numbers (around 80 copies), whereas there was no upper limit of copy number observed for *MIH1*. Although they had different phenotypes, the four genes that showed restricted gene dosage in the Moriya study were all high flux control genes in our experiments, whereas the *mih1* deletion series behaved like wild type in terms of growth rate and cell cycle profile. Both studies show the tight regulation of gene expression for genes that impact on G2/M CDK activity.

This present study also complements the increasing amount of data on the relationship between gene dosage variation and phenotype – in particular, the cataloguing of drug sensitivity/gene CNV ‘fingerprints’ for tumour cells [57], and recent transcriptome studies of yeast strains adapted to different habitats to elucidate gene-environment interactions [58].

### The use of tetraploid series in flux-control analysis

Our work re-establishes the use of tetraploid deletion series as a way of analysing the control of flux (following [8]) and also facilitates the systematic study of gene dosage effects. By varying dosage through a series of clearly resolved (~25% of WT mRNA dosage) steps, we can capture dynamics obscured in gene-deletion studies. Indeed, deleterious phenotypes upon copy-number variation may even be masked by complete gene deletion due to the induction or derepression of alternative pathways [59]. The finer resolution provided by the tetraploid deletion series has allowed us to experimentally characterise the non-linearity of the relationship between gene dosage and the stress response - for example the response of the *HOG1* series to salt treatment – and to begin to understand behaviours previously predicted by dynamic modelling of MAPK pathways (see [60] for a review).

However, a number of experimental refinements could be made to allow more efficient construction of tetraploid series, particularly in the creation of the mating-competent precursor diploids. In this study, we sought to keep our tetraploids isogenic with the strains studied in [6], and therefore did not introduce additional selectable markers during strain construction. Using this method, we isolated the mating-competent diploids at frequencies of 10-20% and 0.5% for the *MAT* ***a***/*MAT* ***a*** and *MATα*/*MATα* strains, respectively. For larger-scale analyses, however, efficiency would be significantly enhanced by the use of selectable markers under the control of the mating-pheromone promoters [61] to isolate mating-competent diploids, and another marker to select tetraploids [27]. A further point to consider is the inherent genome instability of yeast tetraploid strains and to ensure that chromosome loss does not occur during the course of an experiment. We addressed this by minimising the amount of time our cultures spent in stationary phase, an approach that we would recommend in studies using tetraploid series.

### Tetraploid-specific phenotypes

In terms of phenotypic analysis, it should be noted that, in the current study, we measured the growth rate of individual strains during exponential phase in batch culture (~six generations). This is in contrast to our previous work (in which the heterozygous diploid strains were grown in continuous cultures in competition for four days; 30–35 generations); thus, batch culture is a less sensitive method of identifying growth rate variation. This may account for two genes – *HOG1, MIH1* - not showing any growth rate difference relative to WT, in contrast to the diploid result. With this caveat, we now consider the congruence between the predictions of our logical model and the experimentally determined phenotypes. In most modelling studies, deviations between a model’s predictions and the experimental observations contribute to the refinement and improvement of the model. However, in this case, disagreement between experiment and simulation could also result from behaviour unique to the tetraploid cell cycle, which was not found in either the diploid or haploid cell cycles on which the model was based. Where the architecture of the tetraploid cell diverges from that of the diploid/haploid, presenting external (non-cell cycle) perturbations, our model is unable to correctly reproduce the phenotypes observed.

We uncovered two such tetraploid-specific phenotypes in the course of this study. The first is the growth-rate trend of the *SLT2* deletion series, which revealed a tetraploid-specific basal level of activation of the *SLT2* cell wall integrity pathway. We found that *slt2* null tetraploids are severely fitness-compromised even in the absence of cell-wall stress, whereas *slt2* haploids and diploids are completely viable. This is in agreement with the findings of Wu et al. [40] that the decreased ratio of the cell surface area to cell volume in larger cells leads to altered regulation of cell surface components. In that study, *SLT2* mRNA was found to be two-fold more abundant in tetraploids than in haploids [40]. The Slt2 signalling pathway is thought to respond to changes in the plasma membrane when it is either stretched or has altered connections to the cell wall [11]. Thus it may be that this pathway is always activated in tetraploids because of the limited cell surface area and, as a consequence, Slt2p is required constitutively.

Reducing *SLT2* copy numbers to intermediate levels (in the two- and three-copy mutants) could prompt a reduction in this tetraploid-specific basal level of signalling, thereby relaxing the cell-cycle regulation imposed in the wild type. This might account for the increase in growth rate relative to WT that we observed in these intermediate deletion mutants. This growth advantage was not observed in the diploid, supporting the contention that it is indeed a function of tetraploid-specific pathway activation. Previously, three regulatory processes were shown to be essential specifically in tetraploids – homologous recombination, kinetochore function, and spindle chromatid cohesion [27,62], and our findings suggest that the CWI pathway is a fourth essential process.

The *CDC28* series also demonstrated tetraploid-specific phenotypes: reduction of *CDC28* copy number in the tetraploid compromises fitness, whereas we previously found *CDC28* to be haploproficient in the diploid [7]. In the latter case, we attribute *CDC28* haploproficiency (at least, in part) to the reduced activity of cell-cycle checkpoints (in particular, the spindle checkpoint), which are redundant in the absence of a cell cycle defect. In the tetraploid, by contrast, we propose that these checkpoints are *required*, and may not be omitted, such that reduced levels of Cdc28p are disadvantageous to the higher ploidy cell. It has been shown that, without any perturbation, rates of spontaneous DNA damage and syntelic kinetochore attachment are higher in tetraploids than in diploids or haploids [27]. Consequently, regulators of sister chromatid cohesion and the spindle/kinetochore functions are essential in the tetraploid [27,62] and both these pathways explicitly require Cdc28p activity.

### Bimodal behaviour of the *CDC28*-series cell cycle profiles

The most intriguing and, we believe, novel prediction emerging from our modelling is the bimodal transition from G2 arrest, at *CDC28* gene dosages above 25% of wild type, to G1 arrest as the *CDC28* dosage is lowered below this threshold. This predicted behaviour is consistent with a model wherein cyclin-dependent kinase (CDK) activity progressively increases in one cell cycle, and where a higher level of CDK activity is required at the onset of mitosis than at the G1/S transition [63]. It is difficult to determine the mechanism for this because Cdc28p forms many different cell cycle complexes that are each subjected to several levels of regulation. One explanation for the greater requirement for G2/M CDK activity is the different substrate specificities of the G1/S and G2/M CDKs which have been measured in budding yeast. Accordingly, the complex of Cdc28p with the G1/S cyclin, Clb5p, exhibits higher specificity for target proteins (by approximately 100-fold) than does the Cdc28p-Clb2p (G2/M phase) complex [64]. Thus, the threshold level of Cdc28p at which G1/S targets are able to be phosphorylated is lower than for G2/M. Another explanation is that, for G1, it is the level of the G1 cyclins and not Cdc28p that is limiting for G1/S progression. In support of this, Di Talia and colleagues [65] have shown that overexpression of either *CLN2* or *CLN3* makes progression through G1 faster. G2 accumulation therefore occurs whilst the Cdc28p concentration is above the G1/S threshold level, and the phenotype switches to G1 arrest when Cdc28p falls further, *i.e.* to a level beneath that threshold. A third explanation is the presence of G2/M CDK inhibitor Swe1p. Previous work from the Kitano group [56,66] has shown that the gene dosage ratio between both inhibitors and activators at the G2/M transition are tightly maintained in a stable cell cycle. Our results with the *swe1*, *clb2* and *hsl1* deletion series also support this. We predict a lower G2/M requirement for Cdc28p (and likewise Hsl1p and Clb2p) in a background with lower than WT Swe1p levels.

We found that *CDC28* essentiality imposes a limit on the degree to which its mRNA levels could be reduced; using the DAmP allele approach, the limit was *ca.* 19% of WT levels, as discussed above. Hence, in our tetraploid study, we could not perform an experimental test of the model’s predictions for cell-cycle phasing at the lowest levels of *CDC28* dosage. However, our modelling results were confirmed using an inhibitor-specific *cdc28-as1* diploid mutant. This demonstrates that novel, correct, predictions can be made by Boolean modelling of the cell cycle.

## Conclusions

Our work has re-established the use of tetraploid deletion series as a way of analysing the control of flux and provides a new approach to the systematic study of gene dosage effects. By varying dosage through a series of clearly resolved steps, we captured dynamics obscured in previous gene-deletion studies. An existing logical model of the yeast cell cycle was greatly extended by adding a further 30 cell-cycle-related genes that we had previously shown to exert a high degree of control over yeast growth rate. We also added several further self-contained modules to the model (representing the spindle checkpoint, and the DNA damage-response checkpoint), which can be selectively incorporated into simulations. For the majority of strains, the model’s predictions agreed with experimental observations, validating our model and its use for further predictions. Where simulation and experiment diverged, we uncovered both novel tetraploid-specific phenotypes and a switch in the determinative execution point of a key cell-cycle regulator, the Cdc28 kinase, from the G1/S to the S/G2 boundaries. All of this demonstrates that novel, correct predictions can be made by Boolean modelling of the cell cycle.

## Methods

### Cell cycle modelling

Our model builds on that of Fauré *et al.*[23], who adapted an earlier differential model [24] into a multi-valued logical format, thereby removing the requirement for quantitative parameter determination whilst preserving the qualitative molecular relationships and model behaviour. Beginning with this core model, we introduced a further 30 nodes to the original 22 defined by [23]. These represented the HFC genes, connected by approximately 150 new edges (see Additional file 1 for a list of the species added and for an annotated version of the extended model’s Python script). The logical relationships linking in the new nodes were inferred from published data on the wiring of the *S.cerevisiae* cell cycle [25]. Additional modules describing cell cycle checkpoints were developed as described in [9].

### Modelling gene copy number variation

The homozygous deletion of a gene was simulated by setting the node representing the gene to zero within each time-step. In modelling the heterozygous deletions (50% of WT gene dosage, for example), the value of the node is given by the following rubric:

If the logical requirements for node activation are *not* satisfied: Node value = 0

If the logical requirements for node activation *are* satisfied:

Node value = 0 with probability 0.5

Node value = 1 with probability 0.5

The latter was achieved through the use of a (pseudo-) random number generator on the interval (0,1), with the node set to zero for values less than 0.5, and one for values greater than 0.5. Where the node is multi-valued (as is the case for, *e.g.*, *CLB2*), if the node value is not reset to zero, its value is maintained at the level for which the logical requirements are satisfied. Within a time-step, for each occurrence of the node in a logical statement, an independent call was made to the random function to determine the node value within the expression. Similarly, for 0, 25 and 75% of WT gene dosages, the probability of node value being reset to zero is 1, 0.75 and 0.25, respectively.

### Modelling the growth rate

The WT cell cycle was simulated with a period of 31 time-steps, with a complete cycle defined (as in [23]) as the progression from the state representing START through the activation of the CYTOKINESIS variable to level 2, and, finally, the return of the value of MASS, after cytokinesis, to one, which initiates the next START. We defined a complete cytokinesis event as a run of more than two successive time-steps having the CYTOKINESIS variable at level 2. To calculate the growth rate of deletion strains, simulations were run over 10,000 time-steps and the number of complete cytokinesis events counted. Results were averaged over 10,000 simulations. Thus, the relative growth rate of a deletion strain is given by the average number of time-steps to complete its cell cycle divided by the length of the wild-type cycle (31 time-steps).

### Experimental methods

#### Tetraploid strain construction

Tetraploids were derived from the BY4743-based heterozygous and homozygous diploid deletion collections [67]. Diploids were made homozygous at the *MAT* locus by transformation with the pGal-*HO* endonuclease plasmid [68] and pGal-*HO* induction following a protocol from the Haber laboratory [69]. pGal-*HO* expression was induced with 2% galactose for 40 minutes, cells were plated out at a density of 2000 cells per 90 mm plate, and were grown for 20–24 hours. This allowed us to screen a large number of colonies by replica-mating to standard yeast mating-type tester strains, followed by colony PCR to verify the presence of a single *MAT* allele (see Additional file 1: Methods for primers). Using this method, we isolated *MAT* ***a***/*MAT* ***a*** diploids at a frequency of 10-20% and *MATα*/*MATα* diploids at a frequency of 0.5%.

Compatible diploid maters were mixed in liquid YPD medium to allow them to mate and form tetraploids. The WT tetraploid was the product of mating WT *MAT* ***a***/*MAT* ***a*** and WT *MAT* ***α***/*MAT* ***α***; the three-copy tetraploid was constructed by mating a heterozygous diploid with a WT; the two-copy by mating heterozygous diploids of each mating type; the single-copy by mating one homozygous and one heterozygous diploid; and the null mutant (for the non-essential genes) by mating homozygous diploid deletants of each mating type. Single-copy and *cdc28-DAmP* tetraploid strains, and the diploid strain carrying either the wild-type *CDC28* or *cdc28-as1* allele on a single-copy plasmid were created as detailed in Additional file 1: Methods. Tetraploid zygotes were visible after 3–4 hours and were isolated using a micromanipulator (MSM, Singer Instruments). The presence of both *MAT* ***a*** and *MATα* loci was verified by colony PCR, tetraploid DNA content was verified by flow cytometry.

#### Flow cytometry

To analyse DNA content in tetraploid populations, cells were harvested in mid-exponential phase (OD_600_ = 0.4 to 0.5). For the diploid *cdc28-as1* (F88G) strain, cells were grown to exponential phase in YPD, harvested, and the medium replaced with YPD plus the required concentration of inhibitor, and returned to 30C with samples taken at 30 minutes, 1 hour, 2 hours and 3 hours thereafter. Cells were fixed and stained using the method of [70]. We used a CyAn ADP analyzer (Beckman Coulter) and Flow-Jo software (Treestar) to visualise DNA content distribution.

To make a distinction between G2 and S-phase cells within populations having a 2N DNA content, we used multispectral imaging flow cytometry according to a previously published protocol [71], but with the following changes. Sytox-green-stained cells were resuspended in phosphate-buffered saline at a concentration of 10^7^ cells/ml prior to loading them into an Imagestream imaging flow cytometer (Amnis Corporation). 4000 images of each strain were taken and analysed using the IDEAS software package (Amnis Corporation). Cell cycle gates were based on fluorescence, nuclear morphology, and the presence of a bud [71]. For cells with a G2 DNA content, those having a nuclear aspect ratio of 0.7 to 1 were classified as S/G2-phase cells, and those having a ratio below 0.7 as mitotic cells.

#### Measurement of growth rates

Strains were grown in YPD rich medium. In liquid medium, 2 μl of stationary phase cultures (40 to 48 hours) were diluted with 200 μl fresh YPD in 96-well plates and grown at 30°C in a BMG Fluostar Optima plate-reader. OD_595_ measurements were taken every 10 minutes to plot the growth curve, and a curve-fitting algorithm written in R used to calculate the strain minimum doubling time. Each tetraploid strain was grown in triplicate and the average minimum doubling time was calculated. The growth rate (proportional to the inverse of the doubling time) relative to the WT was calculated as the WT average minimum doubling time divided by the average minimum doubling time for each strain.

For solid assays, the required drug or stress agent was added to YPD-agar. Overnight cultures of the tetraploid strains were spotted onto plates using a Singer rotor HDA colony-pinning robot. Plates were incubated at 30C and photographed at 24 and 48 hours and analysed using a custom-written image processing script in MatLab (Mathworks). Colony size for each strain was compared with both WT growth on the same plate and with growth in the absence of drugs. This stressed : unstressed ratio was calculated for every strain on a plate, and the standard deviation of all ratios calculated. Strains having a stress: unstressed ratio greater than two standard deviations from that of the WT were deemed significantly resistant or sensitive. Hierarchical clustering was performed both for the strains, and the different stress treatments, using Cluster 3.0 software [72], and visualised using Java TreeView software. Pairwise correlation coefficients were calculated to determine the similarities between individual gene stress-response profiles.

## Abbreviations

MCA: Metabolic control analysis; WT: Wild-type; HFC: High-flux control; HI: Haploinsufficient; HP: Haploproficient; MAPK: Mitogen-activated protein kinase; CWI: Cell wall integrity; HOG: High osmolarity glycerol; YPD: Yeast peptone dextrose medium; PI: Propidium iodide; RT-PCR: Reverse-transcription polymerase chain reaction; CDK: Cyclin-dependent kinase; DAmP: Decreased abundance by mRNA perturbation.

## Competing interests

The authors declare that they have no competing interests.

## Authors’ contributions

AAA constructed the tetraploid series and performed most of the experiments; MdC constructed the model and performed simulations and the inhibitor-specific and stress-sensitivity experiments; PP measured growth rates on solid media and performed continuous culture experiments; SGO designed the study and wrote the paper together with MdC and AAA. All authors were involved in the interpretation of data. All authors read and approved the final manuscript.

## Supplementary Material

Additional file 1: Table S1 Genes added in this work, to the Fauré *et al.*[23] logical cell cycle model. **Table S2:** List of all strains and plasmids used in this study, including mating-competent diploids and tetraploid deletion mutants. **Table S3:** Primers used in this study. **Figure S1**: mRNA levels in the tetraploid deletion series. **Figure S2:** The effect of ploidy on tolerance to cell wall-specific stressors. Maximum growth rate upon treatment with 20-40μg/mL calcofluor white, and 1-2 M sorbitol, relative to untreated growth, for WT tetraploid, diploid and haploid cells. **Figure S3:** Model predictions for the cell cycle profiles of *SLT2*, *SWE1* and *HOG1*, which are largely unperturbed from the wild type. G1: blue; S/G2: red; M-phase: green. **Figure S4:** Viability of the tetraploid strains, quantified by the degree of uptake of phloxine B and propidium iodide stains. **Figure S5:** Lengths of the cell cycle and G1, S/G2, M phases of the *CDC28*, *CLB2* and *HSL1* tetraploid series. **Figure S6:** Sensitivity of the series of *cdc28* deletion mutants to G1 and G2/M stressors. **Figure S7:** mRNA levels of the downstream DNA damage reporter genes *DUN1, DUN7, RAD54, PLM2* and *RNR3* in *CDC28* deletion mutants, and in the wild type. **Model S1:** Annotated Python script for the Extended Cell Cycle Model. Supplementary references. Click here for file
