# Supplementary material for: Control analysis of the eukaryotic cell cycle using gene copy-number series in yeast tetraploids
Source: BMC Genomics. 2013 Oct 31;14:744. doi: 10.1186/1471-2164-14-744 (PMC3826841; doi:10.1186/1471-2164-14-744)
Supplement: Additional file 1: Table S1 — Genes added in this work, to the Fauré et al.[23] logical cell cycle model. Table S2: List of all strains and plasmids used in this study, including mating-competent diploids and tetraploid deletion mutants. Table S3: Primers used in this study. Figure S1: mRNA levels in the tetraploid deletion series. Figure S2: The effect of ploidy on tolerance to cell wall-specific stressors. Maximum growth rate upon treatment with 20-40μg/mL calcofluor white, and 1-2 M sorbitol, relative to untreated growth, for WT tetraploid, diploid and haploid cells. Figure S3: Model predictions for the cell cycle profiles of SLT2, SWE1 and HOG1, which are largely unperturbed from the wild type. G1: blue; S/G2: red; M-phase: green. Figure S4: Viability of the tetraploid strains, quantified by the degree of uptake of phloxine B and propidium iodide stains. Figure S5: Lengths of the cell cycle and G1, S/G2, M phases of the CDC28, CLB2 and HSL1 tetraploid series. Figure S6: Sensitivity of the series of cdc28 deletion mutants to G1 and G2/M stressors. Figure S7: mRNA levels of the downstream DNA damage reporter genes DUN1, DUN7, RAD54, PLM2 and RNR3 in CDC28 deletion mutants, and in the wild type. Model S1: Annotated Python script for the Extended Cell Cycle Model. Supplementary references. [file 1471-2164-14-744-S1.docx]

**Alcasabas, de Clare, Pir & Oliver**

**Supplementary Information**

**Methods**

**Real Time (RT) PCR to measure transcript levels**

Total RNA was extracted from 10ml exponential-phase cultures (OD600 = 0.4 to 0.6) using standard procedures with Trizol reagent (Invitrogen) and chloroform. Total RNA was quantified by measuring absorbance at 260nm and by visualisation on an agarose gel. cDNA was amplified from approximately 500ng of total RNA using SuperScriptIII (Invitrogen). The resulting cDNA (20µl) was diluted to 400µl with nuclease-free water. 3µl was used for every 19µl RT-PCR reaction.

RT-PCR was performed in a Rotor-Gene 6000 (Corbett Research), all primers and probes are listed in Supplementary Table S3. For the *cdc28* tetraploid and diploid deletion series, transcripts of *ACT1*, *CDC28*, and damage-inducible genes were amplified using reagents and conditions specified by the Rotor-Gene SYBR Green Kit (Qiagen). RT-PCR conditions used were 5 min. initial denaturation at 95°C, followed by 35 cycles of 30 sec each at 95°C, 56°C, and 72°C when fluorescence was measured in the green channel, followed by 10 min. at 72°C, and finally a melting curve where the temperature was raised by 1°C every five sec and fluorescence measured until 99°C. Apart from the expected fluorescence curves during both the PCR and melting steps, we also confirmed that these conditions and primers produced only the expected PCR product by agarose gel electrophoresis (data not shown). No-template controls were used for each set of reactions, and RT-PCR quantitation was also initially tested using different dilutions of cDNA from WT.

For the other gene deletion series, *ACT1* and *kan*MX transcripts were measured in a duplex reaction using reagents and conditions specified by the Rotor-Gene Multiplex Kit (Qiagen), and primers and fluorescent probes listed in Supplementary Table S3. RT-PCR conditions used were 5 min. initial denaturation at 95°C, followed by 35 cycles of 15 sec. each at 95° and 60°C, when fluorescence was measured in both the green (*kan*MX) and yellow (*ACT1*) channels. The target gene for each series was amplified in a separate RT-PCR reaction using the Rotor-Gene SYBR Green Kit (Qiagen) and conditions described above for *CDC28*.

For each strain, RT-PCR using reference *ACT1* primers, was first performed in triplicate to confirm that the cycle threshold (Ct) values for all strains were within 1 cycle. Ct was calculated using the Rotor-Gene 6000 software (Corbett Research). We then performed RT-PCR for each test primer in triplicate, together with reference *ACT1*. The Ct value taken for each primer was the average of the closest two replicates, eliminating the third replicate. Where there was no clear outlier, then the average Ct of all three replicates was taken. To calculate gene transcript concentration relative to that of the WT strain (Supplementary Figure S1), we used the following formula:

**Construction of a *CDC28* dosage series**

The *CDC28* tetraploid dosage series was derived from the heterozygous *CDC28/cdc28* diploid. This strain was transformed with pAA404 (a centromeric plasmid bearing *URA3* and *CDC28*) and sporulated to obtain the *cdc28* haploids AY274B and AY277 in which the null mutation is complemented by the presence of pAA404 (confirmed by the transformants’ sensitivity to 5-fluoro-orotic acid; 5FOA). These two haploids were crossed to obtain the *cdc28/cdc28* diploid. To convert this diploid to MATa/MATa and MATα/MATα diploids (as described in Methods), we swapped pAA404 with pAA402 (a centromeric plasmid bearing *HIS3* and *CDC28*) in order to use pGAL-*HO*, which has *URA3* as the selectable marker. The resulting strains were named AY389 and AY390A (all strains and plasmids are listed in Supplementary Table S2).

The single-copy *CDC28* tetraploids, AY398D_4 and AY398D_14, were constructed by crossing mating-competent diploids AY389 and AY244A. Tetraploids were streaked out to single colonies and replica-plated onto a medium without histidine to identify tetraploids that were cured of plasmid pAA402.

To make *cdc28*-DAmP tetraploids, we first obtained a *cdc28*-DamP diploid by crossing the cdc28-DAmP haploid *MAT****a*** strain (Breslow *et al*., 2008; OpenBiosystems) to AY274B. The resulting single-copy DAmP diploid, AY412A, was converted to a *MAT****a***/*MAT****a*** diploid, strain AY409A with pGAL-*HO* (Materials and Methods). This was then crossed with AY389 and AY249 to obtain tetraploids AY413B/C and AY414, respectively; these strains contain the *cdc28*-DAmP allele.

RT-PCR was performed to determine the *CDC28* expression level in all *CDC28* tetraploid and diploid series (Supplementary Figure S1B and S1C).

**Construction of a strain in which Cdc28p activity can be titrated by an inhibitor**

Haploid *S.cerevisiae* strains in which the native *CDC28* locus was replaced with the *kan*MX cassette, and the loss complemented by the presence of pJU1189 (pRS416::*CDC28*) or pJU1203 (pRS416::*cdc28-as1* (F88G)) were obtained from Stefania Vaga (ETH, Zurich).

These were mated with AY274B to produce a cdc28/cdc28 diploid bearing pAA402 and pJU1189 or pJU1203. Strains were re-streaked onto medium without uracil but with histidine to encourage loss of pAA402. After four rounds of re-streaking, single colonies were selected and loss of pAA402 was confirmed by the lack of growth on medium lacking histidine, and the sensitivity of the pJU1203-bearing strain to treatment with 1nm-PP1.

**Quantification of growth and viability on YPD Plates**

Each tetraploid strain was grown in liquid YPD for 48h, then spotted as eight replicates onto a YPD-agar plate containing 0.001% phloxine B. This was done in a 16x24 format using a RoToR HDA robot (Singer Instruments). After 24h, the plate was scanned against a black background using an Epson Perfection 1240 flatbed scanner and saved as 8-bit RGB jpeg file at 300ppi resolution. Background colour was set to black using ImageJ (http://rsb.info.nih.gov/ij) software. The images were then read using MATLAB (MathWorks) into a three-dimensional matrix of intensities. The first two dimensions correspond to the two dimensions of the image, their size being equal to the pixel size of the image. The third dimension corresponds to the colour channels (RGB), hence its size is three. Coordinates of the colony centres are identified interactively as a function of user-defined centres of the four colonies at the corners of the image. The image is partitioned into diamond-shaped sub-images, the centres of which correspond to the centres of the colonies they contain. Every sub-image was analysed for the size of the colony it contains, pixels brighter than the background of the sub-image were counted and total number of pixels were assigned as the size (area) of the corresponding colony. The backgrounds of the sub-regions were calculated as the mean intensity of the diagonals of the sub-region multiplied by a user-defined constant coefficient; in this case, an optimized coefficient (4.5) was used for black background. For colony size quantification, only the intensities from the blue channel were used.

The average colony size (in pixels) of the 8 replicates, determined using the blue channel, was calculated for each tetraploid strain, and plotted relative to wild-type growth, as well as growth in liquid medium and that predicted by modelling (Figure 3).

For viability measurements, within each colony, data from all three channels were used for quantifying phloxine B dye uptake as an indication of the fraction of dead cells in the colonies. The “redness” of a pixel was calculated as follows: The product of the intensities from the blue, red and inverted green channels (calculated as [255 - intensity of green channel]) were normalized by dividing by the cube of the sum of the intensities from red and blue channel. Average “redness” values from all pixels of the colony were multiplied by 100% to calculate a percent “Redness Index”. The average red index of the 8 replicates per strain were calculated and plotted relative to wild-type growth (Supplementary Figure S4A).

**Viability measurements tetraploid cultures**

To measure the proportion of viable cells within liquid cultures (Supplementary Figure S5B), selected tetraploid strains were grown in liquid YPD to an OD600 of 0.4 to 0.6. 500µl of cultures were centrifuged, resuspended in 0.5mg/ml of propidium iodide, and incubated for 5min to 1h. These were analysed using a CyAn flow cytometer (Beckman Instruments) to count the number of dead cells which are fluorescent in a population of 10,000 cells. The percentage of dead cells (fluorescent in the red channel) were plotted relative to the total number of cells (Supplementary Figure S4B).

**Table S1.** *Extension of the logical model of the cell cycle*

Species added to the Fauré *et al.* (2009) cell-cycle model in this work, and the logical rules governing their Boolean values are listed, along with species having altered logical rules due to the addition of new intermediate nodes

| **Species** | **Logical expression** | **Comment** |
| --- | --- | --- |
|  |  |  |
| sit4 | Mass |  |
| cln3 | mass & !rad53 | DNA damage & consequent *RAD53* activation, halts progression through START |
| bck2 | sit4 |  |
| smbf | ! (clb2==3 & !(sic1 \| cdc6)) & (bck2 \| cln3 \| cln2 \| (clb5 & !sic1)) |  |
| cln2 | smbf & mass |  |
| swi5 | mcm1 & ! (clb2 & !(sic1 \| cdc6)) \| (mcm1 & clb2 & !(sic1 \| cdc6) & (cdc14=1 & !net1) \| (cdc14=2 & !net1=2)) |  |
| mpk1 | !bud |  |
| pp2a | as Faure et al. species 'PP2ACdc55' | Complex of several HFC genes, not resolved in this work |
| sld2 | (clb5 & !sic1) \| ((clb2=2 \| clb2=3) & !(sic1 \| cdc6)) |  |
| cdc45 | sld2 |  |
| mcm | cdc45 & (mcm1 \| dbf4) | MCM complex consisting entirely of HFC genes |
| origin | mcm \| (origin & (clb5=3 \| clb2=3 \| (clb5=1 & !sic1) \| (clb2=1 & !(sic1\|cdc6)) | Firing of origins of replication, as in Faure et al. |
| cdc34 | TRUE | Basal value |
| lte1 | G2 & !spindle | G2 represented by activated Clb2/5 & repression of *SIC1* & *CDC6* |
| tem1 | lte1 |  |
| cdc14 | dbf2 & ccr4 |  |
| dbf4 | clb5 & !sic1 |  |
| esp1 | TRUE |  |
| condensin | rad61 & pds1 | Spindle checkpoint, the presence of the additional HFC species is required for spindle formation & elongation. The components of the condensin complex, all HP, are not resolved in this model |
| cohesin & ctf8 | rad61 & pds1 |  |
| bik1 | G2/M & !cytokinesis |  |
| nuf2 | G2/M & !cytokinesis |  |
| mcm21 | G2/M & !cytokinesis |  |
| nkp2 | G2/M & !cytokinesis |  |
| dma1 | G2/M & !cytokinesis |  |
| ndl1 | G2/M & !cytokinesis |  |
| spindle | (condensin & cohesin & ctf8 & (bik1 & nuf2 & mcm21 & nkp2 & dma1 & ndl1)) \| (spindle & G2) |  |
| mad2 | origin & !spindle |  |
| bub2 | origin & (!spindle \| pp2a=2 \| !cdc5polo) |  |
| clb1 | As Faure et al. species ‘clb2’, with dependencies on Cdc20 removed |  |
| clb2 | As Faure et al. species ‘clb2’ |  |
| bfa1 | origin & (!spindle \| pp2a=2 \| !cdc5polo) |  |
| cdc5 polo | !rad53 & clb2 & !(sic1\|cdc6) & !cdh1 |  |
| pds1 | chk1 \| (mc1 & smbf & !cdc20=2) \| ((mcm1 \| smbf) & !cdh1 & !cdc20) |  |
| rad61 | (mcm1 & smbf & !cdc20=2) \| ((mcm1 \| smbf) & !cdh1 & !cdc20) |  |
| msh2 | ss_damage & rad53 | DNA damage response module; damage (SS or DS) persists until repaired, requiring the presence and correct progression of the repair species |
| mlh1 | ss_damage & rad53 |  |
| rad1 | ss_damage & rad53 \| top1 |  |
| dnl4 | ds_damage & rad53 |  |
| ccr4 | ds_damage & rad53 |  |
| csm3 | ds_damage & rad53 |  |
| rad53 | mec1 & !G2 |  |
| rad9 | ds_damage & G2 |  |
| mec1 | ds_damage \| rad9 |  |
| chk1 | mec1 |  |
| ss_damage | ss_damage & !(msh2 & mlh1 \| rad1) |  |
| ds_damage | ds_damage & ( !(dnl4 & ccr4 & csm3) & cohesin & ctf8 \| !epl1 ) |  |
| top1 | top1 & !(rad1 & cdc45) |  |
| hsl1 | (hsl7 \| epl1) \| (hsl1 & !cdh1) |  |
| hsl7 | (bud & !hog1 & !zds1) \| (hsl7 & !cdh1) |  |

Supplementary Table S2. Plasmids and yeast strains used in this study.

| **Plasmids** | | |  |
| --- | --- | --- | --- |
|  | **pGal-HO** | YCp50 (*URA3*) + *HO* under *GAL1* promoter | Herskowitz and Jensen, 1991 |
|  | **pAA 402** | pRS413 (*HIS3*) + *CDC28* under its own promoter | this study |
|  | **pAA 404** | pRS416 (*URA3*) + *CDC28* under its own promoter | this study |
|  |  |  |  |
| **Haploids of *cdc28*** | | |  |
|  | **AY 274B** | *cdc28∆*::*kan*MX *MAT*α *lys2∆0* *leu2∆0* *his3∆1* *ura3∆0* [pAA404] | this study |
|  | **AY 277** | *cdc28∆*::*kan*MX *MAT****a*** *met15∆0* *leu2∆0* *his3∆1* *ura3∆0* [pAA404] | this study |
|  | **cdc28-DamP** | *cdc28-DAmP* *MAT****a*** *met15∆0* *leu2∆0* *his3∆1* *ura3∆0* | Breslow et al., 2008, OpenBiosystems |
|  |  |  |  |
| **Diploids** | | |  |
| **WT** | **BY4743** | *MAT****a***/*MAT*α *his3∆1*/*his3∆1* *leu2∆0*/*leu2∆0* *met15∆0*/*MET15* *LYS2*/*lys2∆0* *ura3∆0/ura3∆0* | Brachmann et al., 1998 |
|  | **WBY25** | as BY4743, *MAT****a***/*MAT****a*** | this study |
|  | **WBY26** | as BY4743, *MAT*α/*MAT*α | this study |
| **HO** | **AY 282** | as BY4743*, ho∆::kanMX4/HO MAT****a***/*MAT****a*** [pGAL-HO] | this study |
| **HOG1** | **AY 257** | as BY4743*, hog1∆::kanMX4/hog1∆::kanMX4 MAT****a***/*MAT****a*** | this study |
|  | **AY 259** | as BY4743*, hog1∆::kanMX4/hog1∆::kanMX4 MATα/MATα* | this study |
|  | **AY 246A** | as BY4743*, hog1∆::kanMX4/HOG1 MAT****a***/*MAT****a*** | this study |
| **MIH1** | **AY 258** | as BY4743*, mih1∆::kanMX4/mih1∆::kanMX4 MAT****a***/*MAT****a*** | this study |
|  | **AY 382a** | as BY4743*, mih1∆::kanMX4/mih1∆::kanMX4 MATα/MATα* | this study |
|  | **AY 247A** | as BY4743*, mih1∆::kanMX4/MIH1 MAT****a***/*MAT****a*** | this study |
| **SLT2** | **AY 253** | as BY4743*, slt2∆::kanMX4/slt2∆::kanMX4 MAT****a***/*MAT****a*** | this study |
|  | **AY 383A** | as BY4743*, slt2∆::kanMX4/slt2∆::kanMX4 MATα/MATα* | this study |
|  | **AY 242A** | as BY4743*, slt2∆::kanMX4/SLT2 MATa/MATa* | this study |
| **SWE1** | **AY 254** | as BY4743*, swe1∆::kanMX4/swe1∆::kanMX4 MAT****a***/*MAT****a*** | this study |
|  | **AY 255** | as BY4743*, swe1∆::kanMX4/swe1∆::kanMX4 MATα/MATα* | this study |
|  | **AY 243** | as BY4743*, swe1∆::kanMX4/SWE1 MAT****a***/*MAT****a*** | this study |
| **HSL1** | **AY 256** | as BY4743*, hsl1∆::kanMX4/hsl1∆::kanMX4 MAT****a***/*MAT****a*** | this study |
|  | **AY 291A** | as BY4743*, hsl1∆::kanMX4/hsl1∆::kanMX4 MATα/MATα* | this study |
|  | **AY 245A** | as BY4743*, hsl1∆::kanMX4/HSL1 MAT****a***/*MAT****a*** | this study |
| **CLB1** | **AY 251** | as BY4743*, clb1∆::kanMX4/clb1∆::kanMX4 MAT****a***/*MAT****a*** | this study |
|  | **AY 288** | as BY4743*, clb1∆::kanMX4/clb1∆::kanMX4 MATα/MATα* | this study |
|  | **AY 240** | as BY4743*, clb1∆::kanMX4/CLB1 MAT****a***/*MAT****a*** | this study |
| **CLB2** | **AY 250A** | as BY4743*, clb2∆::kanMX4/clb2∆::kanMX4 MAT****a***/*MAT****a*** | this study |
|  | **AY 289A** | as BY4743*, clb2∆::kanMX4/clb2∆::kanMX4 MATα/MATα* | this study |
|  | **AY 239** | as BY4743*, clb2∆::kanMX4/CLB2 MAT****a***/*MAT****a*** | this study |
| **CDC28** | **AY 390A** | as BY4743*, cdc28∆::kanMX4/cdc28∆::kanMX4 MAT****a***/*MAT****a*** [pAA402] | this study |
|  | **AY 389** | as BY4743*, cdc28∆::kanMX4/cdc∆::kanMX4 MATα/MATα* [pAA402] | this study |
|  | **AY 244A** | as BY4743*, cdc28∆::kanMX4/CDC28 MAT****a***/*MAT****a*** | this study |
|  | **AY 249** | as BY4743*, cdc28∆::kanMX4/CDC28 MATα/MATα* | this study |
|  | **AY409A** | as BY4743*, cdc28∆::kanMX4/cdc28-DAmP MAT****a***/*MAT****a*** | this study |
|  | **het CDC28** | as BY4743*, cdc28∆::kanMX4/CDC28* | Winzeler et al., 1999, OpenBiosystems |
|  | **AY 412A** | as BY4743*, cdc28∆::kanMX4/cdc28-DAmP* | this study |
|  | **AY 412B** | as BY4743*, cdc28∆::kanMX4/cdc28-DAmP* | this study |
|  | cdc28-as diploid | as BY4743*, cdc28∆::kanMX4/cdc∆::kanMX4* [pJU1203 (pRS416; cdc28-as1 (F88G))] | this study |
|  | *CDC28* ctrl diploid | as BY4743*, cdc28∆::kanMX4/cdc∆::kanMX4* [pJU1189 (pRS416; CDC28)] | this study |
|  |  |  |  |
| **Tetraploids** | | |  |
| **WT** | **AY 353** | WT tetraploid from WBY25 x WBY26 first isolate – *MAT****a***/*MAT****a***/*MAT*α/*MAT*α *his3∆1*/*his3∆1*/*his3∆1*/*his3∆1* *leu2∆0*/*leu2∆0*/*leu2∆0*/*leu2∆0* *met15∆0*/*met15∆0*/*MET15*/*MET15* *LYS2*/*LYS2*/*lys22∆0*/*lys2∆0* *ura3∆0*/*ura3∆0*/*ura3∆0*/*ura3∆0* | this study |
| **WT** | **AY 354** | as AY353 (WT tetraploid from WBY25 x WBY26 second isolate) | this study |
| **3_HO** | **AY 376A** | as AY353*, ho∆::kanMX4/HO/HO/HO* (comparable to WT in growth rate) | this study |
| **0_HOG1** | **AY 397C** | as AY353*, hog1∆::kanMX4/hog1∆::kanMX4/hog1∆::kanMX4/ hog1∆::kanMX4* | this study |
| **1_HOG1** | **AY 401B** | as AY353*, hog1∆::kanMX4/hog1∆::kanMX4/hog1∆::kanMX4/ HOG1* | this study |
| **2_HOG1** | **AY 342A** | as AY353*, hog1∆::kanMX4/hog1∆::kanMX4/HOG1/HOG1* | this study |
| **3_HOG1** | **AY 343A** | as AY353*, hog1∆::kanMX4/HOG1/HOG1/HOG1* | this study |
| **0_MIH1** | **AY 391D** | as AY353*, mih1∆::kanMX4/mih1∆::kanMX4/mih1∆::kanMX4/ mih1∆::kanMX4* | this study |
| **1_MIH1** | **AY 393B** | as AY353*, mih1∆::kanMX4/mih1∆::kanMX4/mih1∆::kanMX4/ MIH1* | this study |
| **2_MIH1** | **AY 347B** | as AY353*, mih1∆::kanMX4/mih1∆::kanMX4/MIH1/MIH1* | this study |
| **3_MIH1** | **AY 365B** | as AY353*, mih1∆::kanMX4/MIH1/MIH1/MIH1* | this study |
| **0_SLT2** | **AY 387A** | as AY353*, slt2∆::kanMX4/slt2∆::kanMX4/slt2∆::kanMX4/ slt2∆::kanMX4* | this study |
| **1_SLT2** | **AY 386B** | as AY353*, slt2∆::kanMX4/slt2∆::kanMX4/slt2∆::kanMX4/SLT2* | this study |
| **2_SLT2** | **AY 371A** | as AY353*, slt2∆::kanMX4/slt2∆::kanMX4/SLT2/SLT2* | this study |
| **3_SLT2** | **AY 339B** | as AY353*, slt2∆::kanMX4/SLT2/SLT2/SLT2* | this study |
| **0_SWE1** | **AY 402D** | as AY353*, swe1∆::kanMX4/swe1∆::kanMX4/swe1∆::kanMX4 /swe1∆::kanMX4* | this study |
| **1_SWE1** | **AY 403C** | as AY353*, swe1∆::kanMX4/swe1∆::kanMX4/swe1∆::kanMX4/ SWE1* | this study |
| **2_SWE1** | **AY 370B** | as AY353*, swe1∆::kanMX4/swe1∆::kanMX4/SWE1/SWE1* | this study |
| **3_SWE1** | **AY 340A** | as AY353*, swe1∆::kanMX4/SWE1/SWE1/SWE1* | this study |
| **0_HSL1** | **AY 392B** | as AY353*, hsl1∆::kanMX4/hsl1∆::kanMX4/hsl1∆::kanMX4/*  *hsl∆::kanMX4* | this study |
| **1_HSL1** | **AY 384B** | as AY353*, hsl1∆::kanMX4/hsl1∆::kanMX4/hsl1∆::kanMX4/HSL1* | this study |
| **2_HSL1** | **AY 368A** | as AY353*, hsl1∆::kanMX4/hsl1∆::kanMX4/HSL1/HSL1* | this study |
| **3_HSL1** | **AY 359A** | as AY353*, hsl1∆::kanMX4/HSL1/HSL1/HSL1* | this study |
| **0_CLB1** | **AY 400C** | as AY353*, clb1∆::kanMX4/clb1∆::kanMX4/clb1∆::kanMX4/clb1∆::kanMX4* | this study |
| **1_CLB1** | **AY 399B** | as AY353*, clb1∆::kanMX4/clb1∆::kanMX4/clb1∆::kanMX4/CLB1* | this study |
| **2_CLB1** | **AY 346B** | as AY353*, clb1∆::kanMX4/clb1∆::kanMX4/CLB1/CLB1* | this study |
| **3_CLB1** | **AY 344A** | as AY353*, clb1∆::kanMX4/CLB1/CLB1/CLB1* | this study |
| **0_CLB2** | **AY 395C** | as AY353*, clb2∆::kanMX4/clb2∆::kanMX4/clb2∆::kanMX4/ clb2∆::kanMX4* | this study |
| **1_CLB2** | **AY 394A** | as AY353*, clb2∆::kanMX4/clb2∆::kanMX4/clb2∆::kanMX4/CLB2* | this study |
| **2_CLB2** | **AY 349B** | as AY353*, clb2∆::kanMX4/clb2∆::kanMX4/CLB2/CLB2* | this study |
| **3_CLB2** | **AY 358C** | as AY353*, clb2∆::kanMX4/CLB2/CLB2/CLB2* | this study |
| **DaMP_ CDC28_b** | **AY 413B** | as AY353*, cdc28-DAmP/ cdc28∆::kanMX4/cdc28∆::kanMX4/cdc28∆::kanMX4* | this study |
| **DaMP_ CDC28_c** | **AY 413C** | as AY353*, cdc28-DAmP/ cdc28∆::kanMX4/cdc28∆::kanMX4/cdc28∆::kanMX4* | this study |
| **1_CDC28_4** | **AY398D_4** | as AY353*, cdc28∆::kanMX4/cdc28∆::kanMX4/cdc28∆::kanMX4/CDC28* | this study |
| **1_CDC28_**  **_14** | **AY398D_14** | as AY353*, cdc28∆::kanMX4/cdc28∆::kanMX4/cdc28∆::kanMX4/CDC28* | this study |
| **1+DaMP**  **_CDC28_a** | **AY 414A** | as AY353*, cdc28-DamP/ cdc28∆::kanMX4/cdc28∆::kanMX4/CDC28* | this study |
| **2_CDC28** | **AY 369D** | as AY353*, cdc28∆::kanMX4/cdc28∆::kanMX4/CDC28/CDC28* | this study |
| **3_CDC28** | **AY 363A** | as AY353*, cdc28∆::kanMX4/CDC28/CDC28/CDC28* | this study |

**Supplementary Table S3.** *Primers Used in this Study*

| **Primer Name** | **Sequence** |
| --- | --- |
| **To amplify CDC28 and its native promoter from genomic DNA** | |
| **Bam-CDC280-F** | ggatcCGCACGCAGTGTATCAATTT |
| **Sal-CDC28-R** | gtcgacAATGACAGTGCAGTAGCATTTG |
|  |  |
| **Mating type determination** | |
| **MAT alpha-F** | gcacggaatatgggactacttcg |
| **MATa-F2** | gcaaagccttaattccaagg |
| **MAT-R** | agtcacatcaagatcgtttatgg |
|  |  |
| **RT-PCR primers to measure CDC28 mRNA level** | |
| **ACT1-RT-F** | CTGCCGGTATTGACCAAACT |
| **ACT1-RT-R** | CGGTGATTTCCTTTTGCATT |
| **CDC28-RT-F** | CCTCGATTTGGACCTGAAAA |
| **CDC28-RT-R** | ACGATGCAGAATACGGTGTG |
|  |  |
| **RT-PCR primers and probes for ACT1 and KanMX duplex RT-PCR** | |
| **ACT1-GS-F** | ATCATGGTCGGTATGGGT |
| **ACT1-GS-R** | CCGTGTTCAATTGGGTAA |
| **ACT1-probe** | HEX-5’-TCTTGGATTGAGCTTCAT-3’-BHQ2 |
| **KanMX-GS-F** | GCAATCAGGTGCGACAA |
| **KanMX-GS-R** | CATCATTGGCAACGCTAC |
| **KanMX-probe** | FAM-5’-ACAACTCTGGCGCATCG-3’-BHQ1 |
|  |  |
| **RT-PCR primers to measure mRNA of other target genes** | |
| **CLB1-RT-F** | CCAAGGACCATTCTCGGTAA |
| **CLB1-RT-R** | GTCATCGGCTCTCGAAACAT |
| **CLB2-RT-F** | TGGTATCCAACTCCCCAAAA |
| **CLB2-RT-R** | TCGCTGAGGAGGATTCTTGT |
| **HOG1-RT-F** | GATGCCGTAGACCTTTTGGA |
| **HOG1-RT-R** | CGTGGTAAGGAGCCGAATAA |
| **HSL1-RT-F** | TGGTCTCGAAGGGAAAGCTA |
| **HSL1-RT-R** | TCAGGCTTCAGATCACGATG |
| **MIH1-RT-F** | TGGCATCTTCTGCACTATCG |
| **MIH1-RT-R** | TTTCGTCGCCTGTACTCTCA |
| **SLT2-RT-F** | AAGGCGATTGACGTATGGTC |
| **SLT2-RT-R** | CTGGGGGTGTCCCTAAAACT |
| **SWE1-RT-F** | CCAACAGCTCTCCACAAACA |
| **SWE1-RT-R** | CTCGTCCGTGCCGTATAAAT |
|  |  |
| **RT-PCR primers for DNA damage inducible genes** | |
| **RAD54-RT-F** | AAGGTGTTGGTGGGTCTCAG |
| **RAD54-RT-R** | GTACGTCCCTGGCTTTTGAA |
| **PLM2-RT-F** | CAACCGCGATTGTATCTCCT |
| **PLM2-RT-R** | GGGATAAAGGCGTTTGTTGA |
| **dun1-rt-f** | CGCGAAAATCCAAGTCAAGT |
| **dun1-rt-r** | GACTTCGGGCGCTACATAAG |
| **DIN7-RT-F** | TAGCGGAATTTGGAAAGTGG |
| **DIN7-RT-R** | AACGCATAATTGGCGAACTC |
| **RNR3-RT-F** | CCGTCTCAGAATTGGATCGT |
| **RNR3-RT-R** | ATTGTTTCCGTTGGAACTGC |

**Supplementary Figure S1.** *mRNA levels in the tetraploid deletion series*. **a)** Relative abundance of *kanMX* transcript (grey bars) relative to the null strain and of specific genes (blue bars) relative to the WT tetraploid strain AY353. **b-c)** Relative abundance of the *CDC28* transcript (blue bars) in both the *cdc28* tetraploid **(b)** and diploid **(c)** deletion series

**
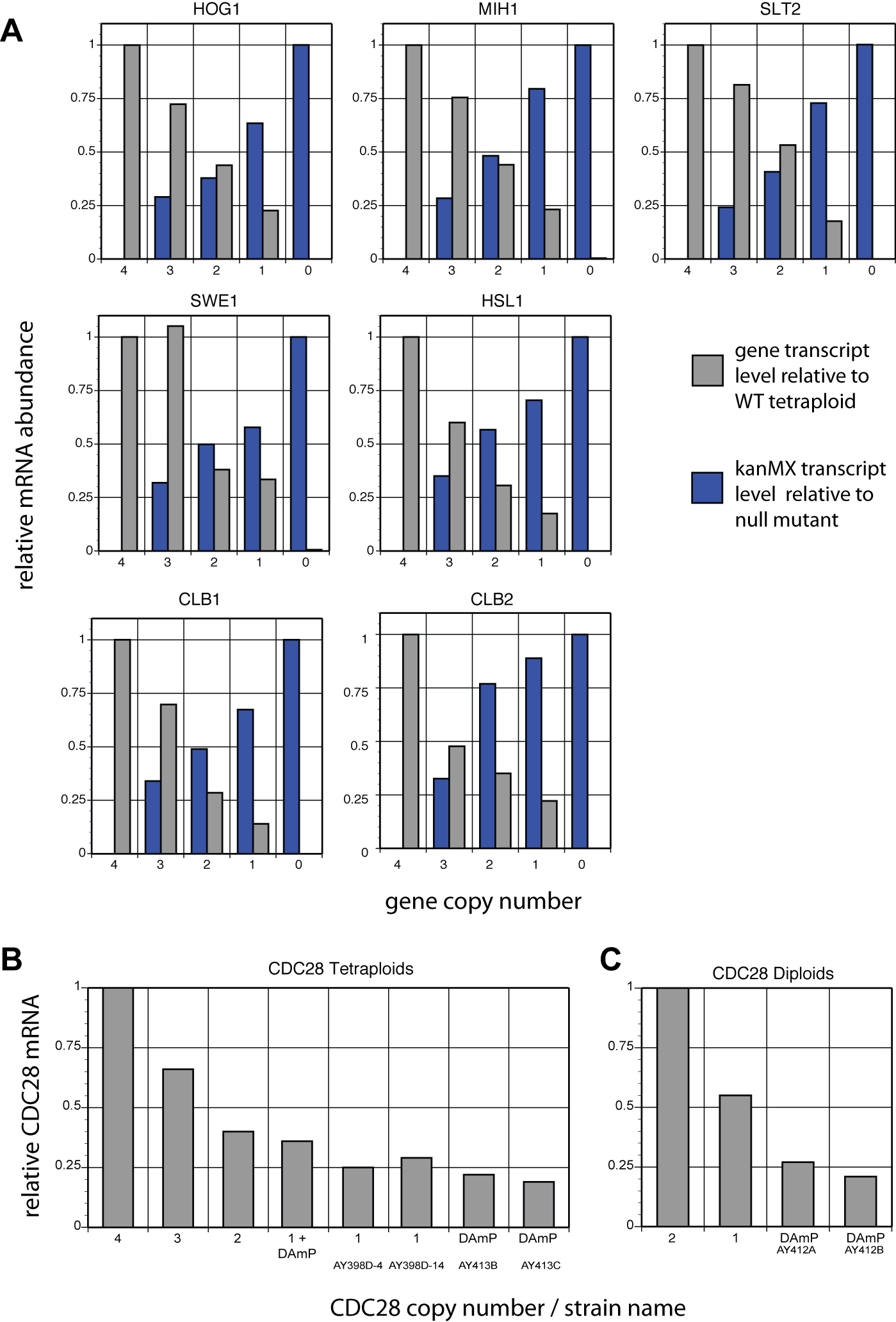
**

**Supplementary Figure S2.** *Effect of ploidy on tolerance to cell wall-specific stressors*.

Maximum growth rate upon treatment with 20-40μg/mL calcofluor white, and 1-2M sorbitol, relative to untreated growth, for WT tetraploid, diploid and haploid cells.


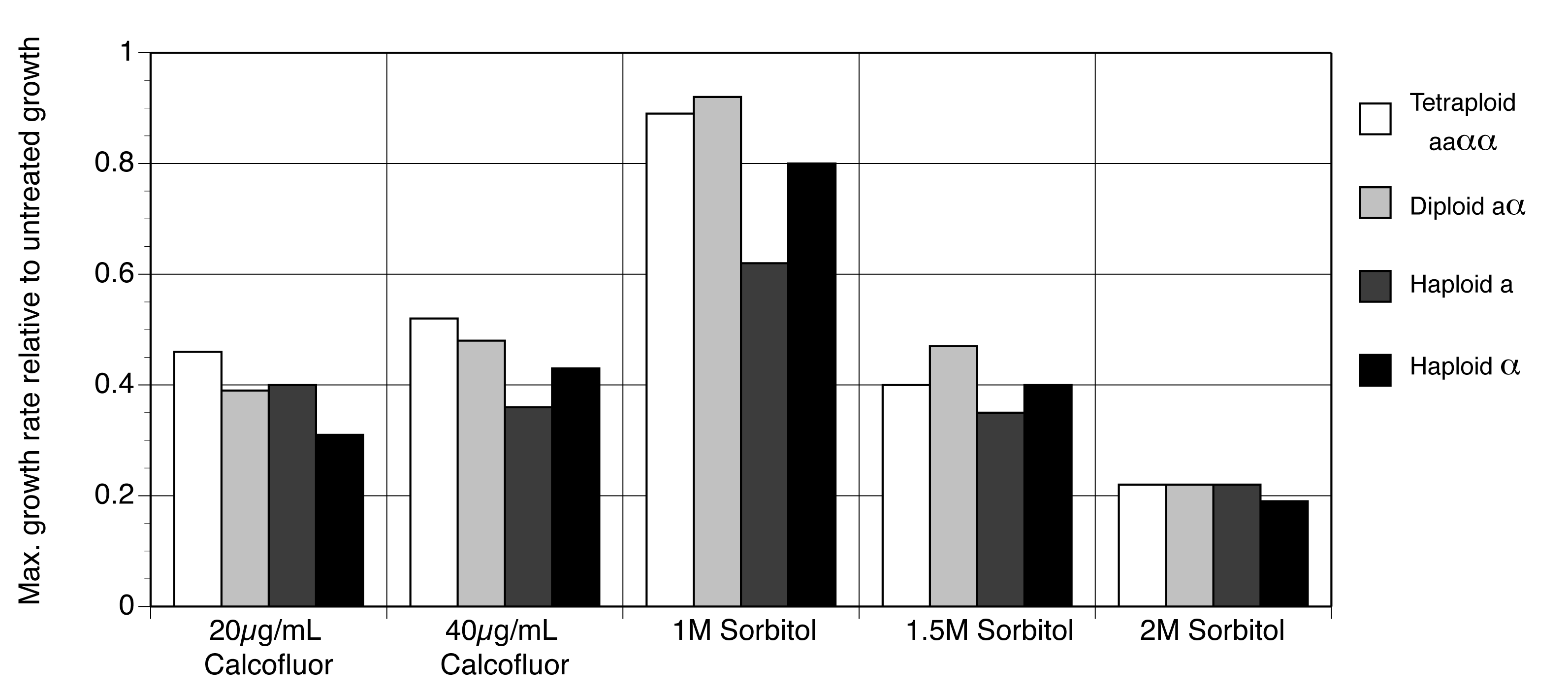


**Supplementary Figure S3.** *Cell-cycle profiles predicted for tetraploid series.*

Model predictions for the cell cycle profiles of *SLT2*, *SWE1*, *HOG1* and *HSL7*, which are largely unperturbed from the wild-type profile. G1: blue; S/G2: red; M-phase: green.

**Supplementary Figure S4.** *Viability of tetraploid strains.*

Proportion of dead cells in tetraploid cultures on YPD agar plates as measured by phloxine-B staining. Pink band indicates range of WT red index (A). Proportion of dead cells of tetraploid strains grown in liquid YPD cultures by flow cytometry (B).

A

B

 **Supplementary Figure S5.** *In vivo cell cycle profiles*

Lengths of the cell cycle and G1, S/G2, M phases of the *CDC28*, *CLB2* and *HSL1* tetraploid series.

**Supplementary Figure S6.** *Response of the CDC28 tetraploid deletion series to G1 and G2/M stressors*

Growth rate relative to WT of the *CDC28* tetraploid deletion series in the presence of 1µg/ml tunicamycin (green filled squares), 2µg/ml tunicamycin (green open squares), and 3µM nocodazole (black triangles).


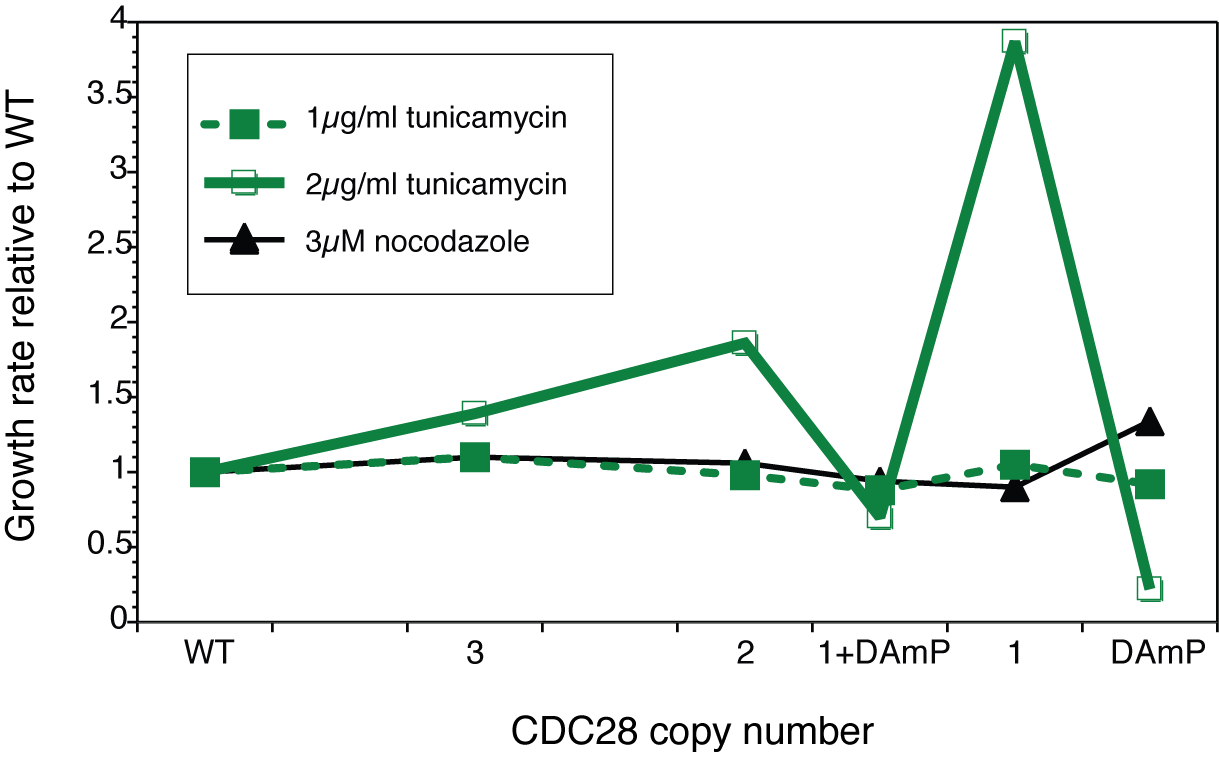
**Supplementary Figure S7.** *Transcript levels of DNA-damage genes.*

mRNA levels of the downstream DNA damage reporter genes *RAD54, PLM2, DUN1, DIN7* and *RNR3* in WT and *cdc28* tetraploid deletion mutants (A) and WT and *cdc28* diploid mutants (B).

**Supplementary Model S1.** *Annotated Python script for the Extended Cell Cycle Model*

#! /usr/bin/python

import random

Degree of knockdown

knockdown_degree = [0,0.25,0.5,0.75,1.]

Genes to be deleted (can be a list of multiple genes, to be deleted individually, or an array of multiples, for multiple-deletion mutants)

target_gene= ['sit4']

for k in range(0,len(target_gene)):

def rsit4(sit4):

if target_gene[k] == 'sit4':

ran = random.random()

if ran>knockdown_degree[i]:

var2=0

else:

var2=sit4

return var2

else:

return sit4

This subfunction (which would used for each gene in the array ‘target_gene’ tests, firstly, that SIT4 is the kth member of the target gene array, and hence that being deleted in the current iteration of the code. Then, a pseudorandom number between 0 and 1 is generated using the NumPy call, and if that number is greater than the current level of knockdown required (i.e. the ith member of the array ‘knockdown_degree’), then the value returned by the subfunction is 0 (i.e., no protein molecule is found). Otherwise, the value 1 is returned (i.e., a molecule of Sit4p is found).

Loop over degree of knockdown (i.e. 4,3,2,1,0 copies respectively)

for i in range(0,len(knockdown_degree)):

for j in range(0,100):

tmax=60

Initialise arrays of values for each gene throughout the cycle

cln3a=[0]*tmax

bck2a=[0]*tmax

smbfa=[0]*tmax

sit4a=[0]*tmax

cln2a=[0]*tmax

clb5a=[0]*tmax

clb2a=[0]*tmax

cln5a=[0]*tmax

yhp1a=[0]*tmax

cdc20a=[0]*tmax

mcm1a=[0]*tmax

mad2a=[0]*tmax

oria=[0]*tmax

spna=[0]*tmax

sic1a=[0]*tmax

rad61a=[0]*tmax

nuf2a=[0]*tmax

mcm21a=[0]*tmax

cdc6a=[0]*tmax

hsl1a=[0]*tmax

swi5a=[0]*tmax

swe1a=[0]*tmax

net1a=[0]*tmax

ck1a=[0]*tmax

cdh1a=[0]*tmax

mpk1a=[0]*tmax

dbf4a=[0]*tmax

cdc34a=[0]*tmax

sld2a=[0]*tmax

cdc45a=[0]*tmax

mcma=[0]*tmax

dbf2_ccr4a=[0]*tmax

cohesin_ctf8a=[0]*tmax

nkp2a=[0]*tmax

mih1a=[0]*tmax

lte1a=[0]*tmax

tem1a=[0]*tmax

cdc15a=[0]*tmax

cdc14a=[0]*tmax

bub2_bfa1a=[0]*tmax

pp2aa=[0]*tmax

cdc5poloa=[0]*tmax

pds1a=[0]*tmax

esp1a=[0]*tmax

buda=[0]*tmax

cytokinesisa=[0]*tmax

condensina=[0]*tmax

bik1a=[0]*tmax

dam1a=[0]*tmax

psa1a=[0]*tmax

top1a=[0]*tmax

epl1a=[0]*tmax

ccr4_csm3a=[0]*tmax

dnl4a=[0]*tmax

rad1a=[0]*tmax

mlh1a=[0]*tmax

msh2a=[0]*tmax

rad53a=[0]*tmax

chk1a=[0]*tmax

mec1a=[0]*tmax

rad9a=[0]*tmax

ss_damagea=[0]*tmax

ds_damagea=[0]*tmax

hsl1a=[0]*tmax

hsl7a=[0]*tmax

dma1a=[0]*tmax

hog1a=[0]*tmax

mcm1a=[0]*tmax

yrb1a= [0]*tmax

zds1a= [0]*tmax

massa=[1]*tmax

rio1a=[1]*tmax

ndl1a=[0]*tmax

Define the initial state of each gene (for those genes in common between the two models, these are the same initial conditions as used in Faure *et al.* 2009)

cln3a[0]=0

bck2a[0]=0

smbfa[0]=0

cln2a[0]=0

cln5a[0]=0

swi5a[0]=0

sic1a[0]=1

cdc6a[0]=1

clb5a[0]=0

mpk1a[0]=1

mih1a[0]=0

hsl1a[0]=0

swe1a[0]=0

clb2a[0]=0

mcm1a[0]=0

mad2a[0]=0

cdc20a[0]=0

cdc5poloa[0]=0

pp2aa[0]=1

bub2_bfa1a[0]=0

lte1a[0]=0

tem1a[0]=0

cdc15a[0]=1

net1a[0]=2

cdc14a[0]=1

cdh1a[0]=1

buda[0]=0

oria[0]=0

spna[0]=0

pds1a[0]=0

esp1a[0]=1

massa[0]=1

cytokinesisa[0]=0

yhp1a[0]=0

sit4a[0]=0

rad61a[0]=0

nuf2a[0]=0

mcm21a[0]=0

ck1a[0]=0

dma1a[0]=0

bik1a[0]=0

ndl1a[0]=0

condensina[0]=0

nkp2a[0]=0

cohesin_ctf8a[0]=0

dbf2_ccr4a[0]=0

mcma[0]=0

sld2a[0]=0

cdc45a[0]=0

cdc34a[0]=0

ds_damagea[0]=0

ss_damagea[0]=0

rad9a[0]=0

mec1a[0]=0

chk1a[0]=0

rad53a[0]=0

msh2a[0]=0

mlh1a[0]=0

rad1a[0]=0

dnl4a[0]=0

ccr4_csm3a[0]=0

epl1a[0]=0

top1a[0]=0

yrb1a[0]=0

psa1a[0]=0

zds1a[0]=0

rio1a[0]=1

Integers to count number of cytokineses within the iteration

cytokinesis_firststep=cytokinesis_secondstep=0

for t in range(1,tmax):

Integers to hold value of the gene at the previous timestep

#g1

cln3=cln3a[t-1]

sit4=sit4a[t-1]

bck2=bck2a[t-1]

smbf=smbfa[t-1]

yhp1=yhp1a[t-1]

cln2=cln2a[t-1]

swi5=swi5a[t-1]

#origin of replication

sic1=sic1a[t-1]

cdc6=cdc6a[t-1]

sld2=sld2a[t-1]

cdc45=cdc45a[t-1]

mcm=mcma[t-1]

cdc34=cdc34a[t-1]

ori=oria[t-1]

#morphogenesis checkpoint

hsl1=hsl1a[t-1]

hsl7=hsl7a[t-1]

swe1=swe1a[t-1]

#g2 phase

clb5=clb5a[t-1]

mpk1=mpk1a[t-1]

mih1=mih1a[t-1]

clb2=clb2a[t-1]

mcm1=mcm1a[t-1]

cdc20=cdc20a[t-1]

epl1=epl1a[t-1]

pp2a=pp2aa[t-1]

net1=net1a[t-1]

cdh1=cdh1a[t-1]

bud=buda[t-1]

pds1=pds1a[t-1]

esp1=esp1a[t-1]

#m phase

lte1=lte1a[t-1]

tem1=tem1a[t-1]

dbf4=dbf4a[t-1]

dbf2_ccr4=dbf2_ccr4a[t-1]

cdc15=cdc15a[t-1]

cdc14=cdc14a[t-1]

#spindle checkpoint

cohesin_ctf8=cohesin_ctf8a[t-1]

rad61=rad61a[t-1]

nuf2=nuf2a[t-1]

condensin=condensina[t-1]

mcm21=mcm21a[t-1]

nkp2=nkp2a[t-1]

dma1=dma1a[t-1]

bik1=bik1a[t-1]

bub2_bfa1=bub2_bfa1a[t-1]

spn=spna[t-1]

ndl1=ndl1a[t-1]

mad2=mad2a[t-1]

cdc5polo=cdc5poloa[t-1]

#dna damage checkpoint

ccr4_csm3=ccr4_csm3a[t-1]

dnl4=dnl4a[t-1]

rad1=rad1a[t-1]

mlh1=mlh1a[t-1]

msh2=msh2a[t-1]

rad53=rad53a[t-1]

chk1=chk1a[t-1]

mec1=mec1a[t-1]

rad9=rad9a[t-1]

ss_damage=ss_damagea[t-1]

ds_damage=ds_damagea[t-1]

top1=top1a[t-1]

#mass and cytokinesis

cytokinesis=cytokinesisa[t-1]

mass=massa[t-1]

hog1=hog1a[t-1]

yrb1=yrb1a[t-1]

psa1=psa1a[t-1]

zds1=zds1a[t-1]

rio1=rio1a[t-1]

Beginning of the logical model – rules as defined in the Supplementary Information

#g1 phase

sit4a[t] = int(bool(mass))

cln3a[t] = int(bool(mass and not rad53))

bck2a[t] = int(bool(rsit4(sit4)))

smbfa[t] = int(bool(not (clb2==3 and not (sic1 or cdc6)) and (bck2 or cln3 or cln2 or (clb5 and not sic1))))

if not smbf and clb2:

smbfa[t]=0

cln2a[t] = int(bool((smbf and mass)))

swi5a[t] = int(bool((mcm1 and not (clb2 and not (sic1 or cdc6))) or (mcm1 and clb2 and not (sic1 and cdc6) and ((cdc14==1 and not net1) or (cdc14==2 and not net1==2)))))

#g2 phase

if bool(cdc20 and smbf and mass):

clb5a[t]=1

if bool(not cdc20 and mass and smbf):

clb5a[t]=2

mpk1a[t] = int(bool(not bud))

if (mpk1 and clb2 and not (sic1 or cdc6)) or (not mpk1 and (not clb2 or sic1 or cdc6)):

mih1a[t] = 1

if mih1 and not mpk1 and clb2 and not (sic1 or cdc6):

mih1a[t] = 2

if ((mass==1 and ((swe1==1 and not mih1==2) or (swe1==2 and mih1==1))) or (mass and swe1==2 and not mih1)) and \

not cdh1 and (not cdc20 or (cdc20==2 and mcm1)):

clb2a[t] = 1

if clb2 and ((mass==1 and (not swe1 or mih1==2)) or (mass==2 and (not swe1==2 or mih1))) and not cdh1 and ((not cdc20 and not mcm1) or (cdc20==2 and mcm1)):

clb2a[t] = 2

if clb2==2 and not clb2a[t]==2:

clb2a[t] = 1

if (clb2==2 or clb2==3) and ((mass==1 and (not swe1 or mih1==2)) or (mass==2 and (not swe1==2 or mih1))) and not cdh1 and not cdc20==2 and mcm1:

clb2a[t] = 3

if not (not esp1 or (esp1==1 and pds1)):

pp2aa[t] = 1

if pp2a and not esp1 or (esp1==1 and pds1):

pp2aa[t] = 2

mcm1a[t] = int(bool((clb2==2 or clb2==3) and not (sic1 or cdc6)))

#origin of replication

if (not cdc14 or (cdc14==1 and net1) or ((cdc14==2 or cdc14==3) and net1==3)) and not swi5 and \

(not ((clb2 and not (sic1 or cdc6)) or (clb5 and not sic1) or cln2 or ((clb2 or clb5) and \

(cln3 or bck2)) or \

(clb5 and clb2) or (clb5==3 and bck2))):

sic1a[t] = 1

elif (not cdc14 or (cdc14==1 and net1) or ((cdc14==2 or cdc14==3) and net1==3)) and swi5 and \

not ((clb2==3 and not (sic1 or cdc6)) or (((clb2 and not (sic1 or cdc6)) or \

(clb5 and not sic1)) and ((cln2 and (cln3 or bck2)) or \

(cln3 and bck2))) or \

(((clb2 and clb5) or clb2==3 or clb5==3) and cln2 and (cln3 or bck2)) or \

(clb2==3 and clb5==3)):

sic1a[t] = 1

elif ((cdc14==1 and (not net1)) or (cdc14==2 and (not net1 or net1==1))) and (not swi5) and \

not ((clb2 and (not (sic1 or cdc6))) or (clb5 and not sic1) or cln2):

sic1a[t] = 1

elif ((cdc14==1 and not net1) or (cdc14==2 and (not net1 or net1==1))) and swi5 and \

not (((clb5 and clb2 and not (sic1 or cdc6) and cln2 and cln3 and bck2) or \

(clb2==3 and not (sic1 or cdc6))) and (clb5 or cln2 or (cln3 and bck2))):

sic1a[t] = 1

elif (cdc14==3 and (not net1==3) and not swi5 and not (clb5 and clb2 and cln2 and cln3 and bck2)):

sic1a[t] = 1

elif (cdc14==3 and (not net1==3) and swi5 and not (clb5 and clb2 and cln2 and cln3 and bck2 \

and not sic1 and not (sic1 or cdc6))):

sic1a[t] = 1

if (not cdc14 or (cdc14==1 and net1) or ((cdc14==2 or cdc14==3) and net1==3)) and not swi5 and \

(not ((clb2 and not (sic1 or cdc6)) or (clb5 and not sic1) or cln2 or ((clb2 or clb5) and \

(cln3 or bck2)) or \

(clb5 and clb2) or (clb5==3 and bck2))):

cdc6a[t] = 1

elif (not cdc14 or (cdc14==1 and net1) or ((cdc14==2 or cdc14==3) and net1==3)) and swi5 and \

not ((clb2==3 and not (sic1 or cdc6)) or (((clb2 and not (sic1 or cdc6)) or \

(clb5 and not sic1)) and ((cln2 and (cln3 or bck2)) or \

(cln3 and bck2))) or \

(((clb2 and clb5) or clb2==3 or clb5==3) and cln2 and (cln3 or bck2)) or \

(clb2==3 and clb5==3)):

cdc6a[t] = 1

elif ((cdc14==1 and (not net1)) or (cdc14==2 and (not net1 or net1==1))) and (not swi5) and \

not ((clb2 and (not (sic1 or cdc6))) or (clb5 and not sic1) or cln2):

cdc6a[t] = 1

elif ((cdc14==1 and not net1) or (cdc14==2 and (not net1 or net1==1))) and swi5 and \

not (((clb5 and clb2 and not (sic1 or cdc6) and cln2 and cln3 and bck2) or \

(clb2==3 and not (sic1 or cdc6))) and (clb5 or cln2 or (cln3 and bck2))):

cdc6a[t] = 1

elif (cdc14==3 and (not net1==3) and not swi5 and not (clb5 and clb2 and cln2 and cln3 and bck2)):

cdc6a[t] = 1

elif (cdc14==3 and (not net1==3) and swi5 and not (clb5 and clb2 and cln2 and cln3 and bck2 \

and not sic1 and not (sic1 or cdc6))):

cdc6a[t] = 1

sld2a[t] = int(bool((clb5 and not sic1) or ((clb2==2 or clb2==3) and not (sic1 or cdc6))))

cdc45a[t] = int(bool(rsld2(sld2)))

mcma[t] = int(bool(cdc45 and (mcm1 or dbf4)))

oria[t] = int(bool(rmcm(mcm) or (ori and (clb5==3 or clb2==3 or (clb5==1 and not sic1) or (clb2==1 and not (sic1 or cdc6))))))

cdc34a[t] = 1

#m phase

lte1a[t] = int(bool(((clb2==2 or clb2==3) and not (sic1 or cdc6)) or spn))

tem1a[t] = int(bool(lte1))

cdc15a[t] = int(bool(not (clb2==3 and not (sic1 or cdc6)) or (cdc14 and not net1)))

cdc14a[t] = int(bool(dbf2_ccr4))

dbf4a[t] = int(bool(clb5 and not sic1))

if not mad2 and mcm1 and not (clb2 and not (sic1 or cdc6)):

cdc20a[t]=1

if not mad2 and mcm1 and clb2==3 and not (sic1 or cdc6):

cdc20a[t]=1

if cdc20 and not mad2 and mcm1 and clb2 and not (sic1 or cdc6):

cdc20a[t]=2

if ((cdc14 and net1 and not (clb2 and not (sic1 or cdc6)) and not pp2a) or (pp2a==1 and ((not cdc14 and clb2==2 and not (sic1 or cdc6)) or \

(cdc14 and clb2==2 and not (sic1 or cdc6))))) \

and not (((cdc15==1 and tem1) or cdc15==2) and not rbub2_bfa1(bub2_bfa1)):

net1a[t] = 1

if ((((cdc14 and not net1) or pp2a) and not (clb2 and not (sic1 or cdc6))) or pp2a==2) and not (((cdc15==1 and tem1) or cdc15==2) and not rbub2_bfa1(bub2_bfa1)):

net1a[t] = 2

cdc14a[t] = 1

if (not cdc14 or (cdc14==1 and net1) or ((cdc14==2 or cdc14==3) and net1==3)) and not ((clb2 and not (sic1 or cdc6))\

or (clb5 and not sic1) or (cln3 and cln2)):

cdh1a[t]=1

if ((cdc14==1 and not net1) or (cdc14==2 and not net1==3)) and not ((clb5 and not sic1 and cln3 and ((clb2 and not cdc6) or cln2)) \

or (clb2==3 and not (sic1 or cdc6) and cln3 and cln2)):

cdh1a[t]=1

if cdc14==3 and not net1==3 and not ((clb5 and not sic1 and cln3 and clb2 and not cdc6 and cln2) or (clb2==3 and not (sic1 or cdc6) and cln3 and cln2)):

cdh1a[t]=1

buda[t] = int(bool((cln2 or (clb5 and not sic1)) and not cytokinesis==2))

esp1a[t] = 1

#spindle checkpoint

condensina[t] = int(bool(rad61 and pds1))

cohesin_ctf8a[t] = int(bool(rad61 and pds1))

bik1a[t] = int(bool(clb2==3 and not (sic1 or cdc6) and not cytokinesis==2))

nuf2a[t] = int(bool(clb2==3 and not (sic1 or cdc6) and not cytokinesis==2))

mcm21a[t] = int(bool(clb2==3 and not (sic1 or cdc6) and not cytokinesis==2))

nkp2a[t] = int(bool(clb2==3 and not (sic1 or cdc6) and not cytokinesis==2))

dma1a[t] = int(bool(clb2==3 and not (sic1 or cdc6) and not cytokinesis==2))

ndl1a[t] = int(bool(clb2==3 and not (sic1 or cdc6) and not cytokinesis==2))

spna[t] = int(bool(((condensin and cohesin_ctf8) and ((rbik1(bik1) and nuf2 and mcm21 and rnkp2(nkp2) and rdma1(dma1) and rndl1(ndl1)))) or \

(spn and (clb2==3 or clb2==2 and not (sic1 or cdc6)))))

mad2a[t] = int(bool(ori and not spn))

bub2_bfa1a[t] = int(bool(ori and (not spn or pp2a==2 or not cdc5polo)))

cdc5poloa[t] = int(bool(not rad53 and clb2 and not (sic1 or cdc6) and not cdh1))

pds1a[t] = int(bool((mcm1 and smbf and not cdc20==2) or ((mcm1 or smbf) and not cdh1 and not cdc20)))

rad61a[t] = int(bool((mcm1 and smbf and not cdc20==2) or ((mcm1 or smbf) and not cdh1 and not cdc20)))

#dna damage checkpoint

msh2a[t] = int(bool(ss_damage and rad53))

mlh1a[t] = int(bool(ss_damage and rad53))

rad1a[t] = int(bool(ss_damage and rad53 or rtop1(top1)))

dnl4a[t] = int(bool(ds_damage and rad53))

ccr4_csm3a[t] = int(bool(ds_damage and rad53))

rad53a[t] = int(bool(mec1 and not ((clb2==2 or clb2==3) and not (sic1 or cdc6))))

rad9a[t] = int(bool(ds_damage and ((clb2==2 or clb2==3) and not (sic1 or cdc6))))

mec1a[t] = int(bool(ds_damage or rad9))

chk1a[t] = int(bool(mec1))

ss_damagea[t] = int(bool(ss_damage and not ((rmsh2(msh2) and rmlh1(mlh1)) or rrad1(rad1))))

ds_damagea[t] = int(bool(ds_damage and ( not (rdnl4(dnl4) and rccr4_csm3(ccr4_csm3)) and cohesin or not epl1)))

top1a[t] = int(bool(rtop1(top1) and not (rrad1(rad1) and cdc45)))

#morphogenesis checkpoint

hsl1a[t] = int(bool((hsl7 or epl1) or (hsl1 and not cdh1)))

hsl7a[t] = int(bool((bud and not rhog1(hog1) and not zds1) or (hsl7 and not cdh1)))

if smbf and ((clb2 and not (sic1 or cdc6) and not hsl1 and not hsl7) or ((hsl1 or hsl7) and not (clb2==2 or clb2==3))):

swe1a[t] = 1

if swe1 and smbf and not (hsl1 or hsl7 or ((clb2==2 or clb2==3) and not (sic1 or cdc6))):

swe1a[t] = 2

#mass and cytokinesis

These logical conditions determine the outcome of the cycle – if the conditions (i.e. large amounts of G2 cyclins, no g1 degraders) are true, then start cytokinesis

if bool(mass and (clb2==2 or clb2==3) and not (sic1 or cdc6)):

cytokinesisa[t] = 1

if bool(massa[t-5] and ((clb2==1 and cytokinesis) or (not clb2==2 and cytokinesis and (sic1 or cdc6)))):

cytokinesisa[t] = 2

if cytokinesis==2 and not cytokinesisa[t]==2:

cytokinesisa[t] = 1

if cytokinesis==2:

massa[t]=0

if massa[t]==2 and not (massa[t-1]==2):

cytokinesis_secondstep+=1

elif massa[t] ==1 and massa[t-1]==0:

cytokinesis_firststep+=1

**References:**

Brachmann CB, Davies A, Cost GJ, Caputo E, Li J, Hieter P, Boeke JD (1998) Designer deletion strains derived from *Saccharomyces cerevisiae* S288C: a useful set of strains and plasmids for PCR-mediated gene disruption and other applications. *Yeast* **14**:115-32.

Herskowitz I, Jensen RE (1991) Putting the HO gene to work: practical uses for mating-type switching. *Methods Enzymol.* **194**: 132-146

Winzeler EA, Shoemaker DD, Astromoff A, Liang H, Anderson K *et al*., (1999) Functional characterization of the *S.cerevisiae* genome by gene deletion and parallel analysis. *Science* **285**: 901–906.
